# Physical activity for people with dementia: a scoping study

**DOI:** 10.1186/1471-2318-13-129

**Published:** 2013-11-26

**Authors:** Alison Bowes, Alison Dawson, Ruth Jepson, Louise McCabe

**Affiliations:** 1 School of Applied Social Science, University of Stirling, Stirling, Scotland FK9 4LA, UK; 2 Scottish Collaboration for Public Health Research and Policy, University of Edinburgh, 20 West Richmond Street, Edinburgh, Scotland EH8 9DX, UK

**Keywords:** Physical activity, People with dementia, Services, Realist review

## Abstract

**Background:**

This scoping study aimed to identify how physical activity may benefit people with dementia; how and/or if current service provide these benefits; and what support they need to do so.

**Methods:**

Methods included an evidence review using literature; mapping current service provision through a survey; and in-depth interviews with a sample of service providers.

**Results:**

The 26 studies included in the review indicated the potential effectiveness of physical activity for people with dementia, including improvements in cognition and mood, behaviour and physical condition. Mechanisms of action and the link with outcomes were poorly defined and implemented.

The mapping survey and related interviews showed that service providers were delivering a range of services broadly consistent with the scientific evidence. They tended to take a holistic view of possible benefits, and focused on enjoyment and well-being, more than specific cognitive, physical and behavioural outcomes highlighted in literature. Service providers needed more evidence based information and resources to develop services and realise their potential.

**Conclusion:**

Despite potential benefits demonstrated in literature and practice, there is a need for further research to optimise interventions and to consider some neglected issues including delivery at home and in communities; impacts for carers; physical activities through ADLs; and individual needs. Studies are needed which take a more holistic approach to the effects of physical activity, and outcomes should be broader and include mental health and wellbeing.

## Background

Physical activity has been identified as relevant to many health outcomes, including: cardiorespiratory health (coronary heart disease, cardiovascular disease, stroke and hypertension); metabolic health (diabetes and obesity); musculoskeletal health (bone health, osteoporosis); cancer (breast and colon cancer); functional health and prevention of falls; and depression [1]. Physical inactivity is the fourth leading global risk for mortality, estimated to be responsible for 5.5% of deaths (3.2 million people) globally in 2004, rising to 7.7% of deaths in higher-income countries [1]. Physical activity is defined as *‘any bodily movement produced by skeletal muscles that results in energy expenditure .... and can be categorised into occupational, sports, conditioning, household, or other activities’*[2]. It should be distinguished from ‘physical exercise’ which is planned, structured, and repetitive and has as an objective of improving or maintaining physical fitness [2].

Dementia is an umbrella term for a number of progressive disorders, of which Alzheimer’s Disease (AD) is the most common, that affect memory, thinking, behaviour and ability to perform everyday activities. Prevalence of dementia is difficult to establish or estimate with certainty, and estimates are affected by differences in study design, disease definition, diagnostic criteria thresholds, and calculation methods e.g. [3,4]. The World Alzheimer Report 2009 [5], which used a meta-analysis of prevalence studies, estimated that globally 35.6 million people aged over 60 (4.7% of that age group) would be living with dementia in 2010, rising to 115.4 million people by 2050.

Physical activity may benefit people with dementia in several ways, though evidence is currently sparse and scattered. Several proposed hypotheses exist to explain how physical activity impacts on dementia [6]. These include a vascular hypothesis; a neurochemical hypothesis; cognitive reserve hypothesis; stress hypothesis; and functional hypothesis. With the exception of the last, all have been derived from animal models. Studies of physical activity programmes with people with dementia suggest multiple benefits including improved cognition [7], activities of daily life and independence [8], functional ability, and mental health [9]. It is likely that social benefits can be significant: for example, if physical activity is undertaken in a group, it can increase social networks and reduce feelings of loneliness and isolation, known to be issues for many people with dementia [10]. The type of physical activity may also be important; for example walking outdoors may help re-establish a connection with nature and the local community; dancing may provide enjoyment and feelings of wellbeing. Physical activity is also likely to have physical health benefits, helping maintain a normal lifestyle and reducing the risk of other disease (e.g. heart disease).

A Cochrane review [11] found there is currently insufficient evidence (only four randomised trials) of the effectiveness of physical activity programmes in managing or improving cognition, function, behaviour, depression, and mortality in people with dementia. That review [11], p16 suggests that ‘further research is necessary to identify the optimal physical activity modalities particularly in terms of frequency, intensity, and duration for persons with different types and severity of dementia’.

One limitation of reviews which assess the effectiveness of physical activity and behavioural change interventions (such as Cochrane reviews) is that most do not describe the intervention in enough detail. It is therefore difficult to ascertain whether it has a coherent theoretical basis and underlying mechanism of action, determine the effective (or ineffective) components, or assess the context in which it is undertaken. Without clarity regarding the components, it is difficult to faithfully replicate effective interventions and challenging to identify techniques contributing to effectiveness across interventions [12]. All these aspects are important for researchers and practitioners aiming to develop and evaluate complex interventions [13]. Furthermore, little attention is paid to evidence from services currently providing these interventions. Current services may offer data to help understand what may work (or not work), for whom, and in what context. Combining research findings with data from services can enable a broader understanding and help in the development of future interventions.

## Methods

The study had three elements. Firstly, a review of the literature using some principles of a ‘realist’ review [14,15] aimed to identify how physical activity may be of benefit to people with dementia. Secondly, current physical activity services for people with dementia across the UK were mapped using an on-line survey; and thirdly, follow up interviews with service providers explored how and/or if current services provide these benefits to people with dementia, and what support service providers might need. Together, the three elements of data collection and analysis were aimed at producing a rounded perspective on physical activity for people with dementia which drew on both the scientific literature and the activities and experiences of practitioners. Ethical approval for the research was given by the Ethics Committee of the School of Applied Social Science, University of Stirling, in compliance with the Economic and Social Research Council’s Framework for Research Ethics.

### Literature review

**Figure 1:**
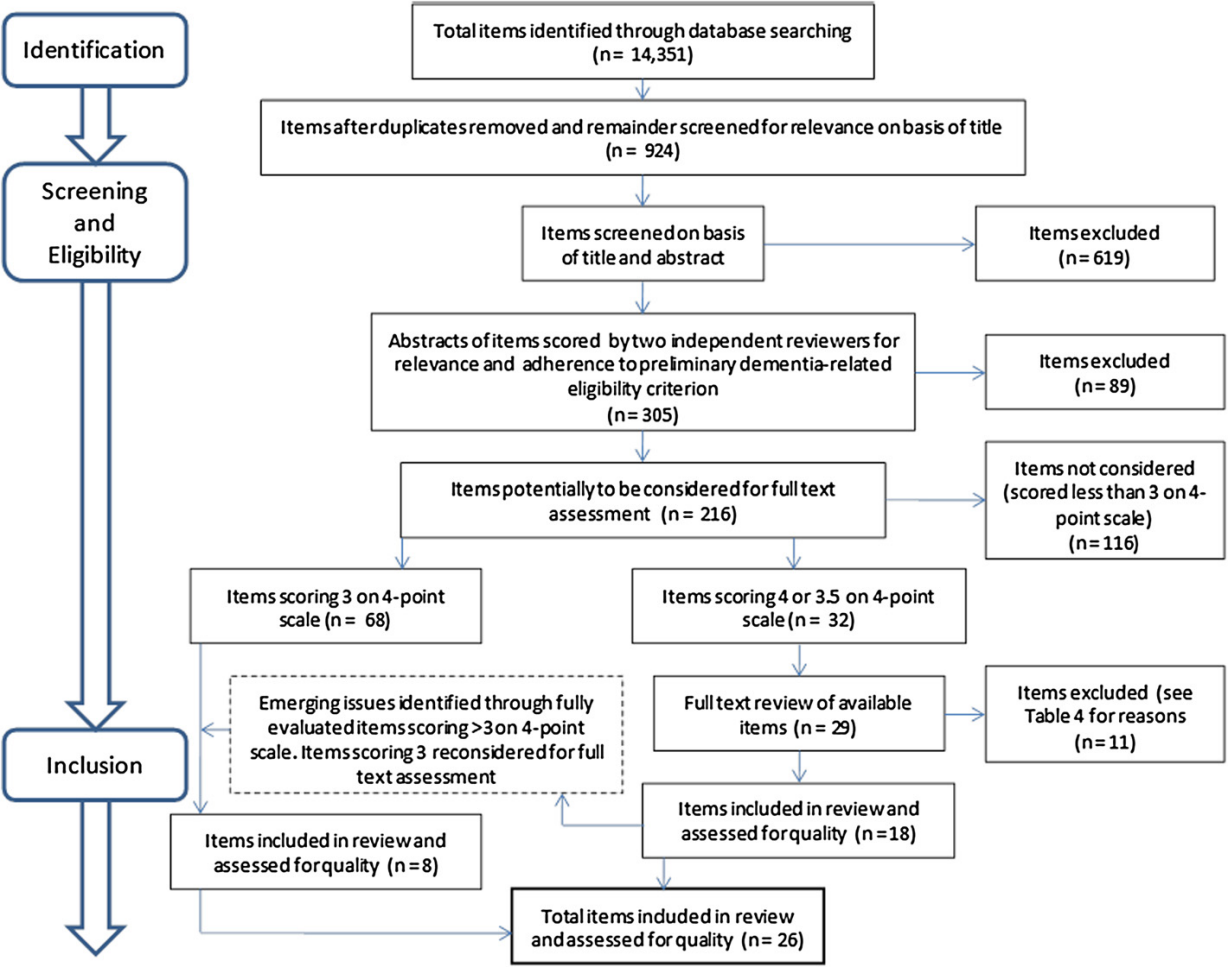
**Flow chart of literature review process (adapted from PRISMA flow diagram **[16]** (Moher et al. 2009).**

The aim of the literature review, following Pawson et al’s [14] recommendations, was to identify and evaluate evidence of how and why physical activity interventions ‘work (or don’t work) in particular contexts or settings’ [14], p5 for people with dementia. Figure 1 summarises the reviewing process.

The Additional file 1: Tables S1, S2, S3, S4, S5, S6 provides more details of the specific methods used in the literature review including search terms, bibliographic databases used, the scoring system used to ascertain item relevance and scores achieved, and a PICO (Populations, Interventions, Comparisons, Outcomes) table of full text items included in the review, which also includes the quality assessments. Criteria for inclusion were: specific inclusion of people with dementia or cognitive impairment; suggesting or explaining mechanisms of action for benefitting from physical activity (physiological, psychological or social); describing or evaluating a specific form of physical activity, rather than referring to physical activity in general; and identifying a specific research study or reviewing a collection of studies.

Two reviewers independently rated and scored the studies and following the process of selection, 26 were included in the final review. Item details and assessments were recorded using a proforma incorporating research design-specific quality assessment templates based on Report no 4, Centre for Reviews and Dissemination (CRD) [17], Cochrane Effective Practice and Organisation of Care (EPOC) group checklists [18] and, as appropriate, Critical Appraisal Skills Programme (CASP) assessment criteria [19]. In cases of disagreement, a third member of the team reviewed the item to arrive at a consensus view.

### The survey

Potential respondents for the on-line survey were recruited through electronic mailing lists with approximately 12,000 contacts including the Dementia Services Development Centre (DSDC) list of people with interests in dementia services and through the Physical Activity Health Alliance (PAHA). These networks represent a wide range of organisations, services providers, professionals, carers and people with dementia across the UK and beyond. Fifty-one people expressed an interest in taking part in the research and were sent the on-line survey, which was directed at those involved in delivering physical activity interventions for people with dementia. The survey was also sent to around 40 others identified as interested in physical activity and dementia following attendance at relevant courses run by the DSDC. Recipients were sent second and third reminder emails and also invited to forward the survey to other relevant parties. SurveyMonkey was used to conduct the survey, and respondents were assured of anonymous and confidential responses. Respondents were invited to volunteer for interview, and the sample for the interviews was drawn from these survey respondents. We estimated that we would receive 75 responses: in the event, 73 usable responses were received. The survey was designed to gain an overview of the activities used, the settings and methods of delivery, the participants, and the thinking behind the interventions (their theoretical basis). The sampling process was clearly non-random and this is a limitation: however, the aim of the survey was exploratory, and in presenting the results, we have described the characteristics of respondents.

### Interviews

The telephone interviews sought to improve understanding of physical activity interventions from the points of view of those delivering them, following up issues that emerged from the survey. Twelve interview participants were selected from 41 volunteers to include a range of activities in different settings and locations, including several who spoke about more than one intervention. The aim of this non-probability sampling process was to maximise the range of coverage in the interviews and gain in-depth knowledge of examples of programmes, their challenges and facilitators. The interviews, which were transcribed and analysed thematically, looked in more detail at the background to the programmes and the thinking behind them, their histories and rationales, their sustainability and the challenges and problems encountered by service providers.

## Results

### Literature review

Our quality assessments indicate few (five) high quality studies in any research paradigm: two are Randomised Controlled Trials (RCTs) [20,21], two, systematic literature reviews [22][23] and one, a controlled feasibility study [24]. Eleven studies were graded medium or medium plus quality, and the remaining ten were of low or low plus quality. In the discussion which follows, we have focused on the higher quality evidence, but are also mindful, following realist review principles [14], that lesser quality studies may nevertheless provide suggestive insights. The table below summarises the methodologies used and the quality assessments of the studies (full details are given in the Additional file 1: Table S6, and conclusions in Additional file 1: Table S7) Table 1.

**Table 1:** Methodologies and quality assessments of studies

Methodology*	**Number**	**High**	**Medium**	**Low**
Qualitative study	1	0	0	1
Randomised controlled trial (RCT)	9	2	6	1
Controlled clinical trail (CCT)	4	0	3	1
Controlled before and after study (CBA)	3	0	0	3
(Systematic) literature review	3	2	0	1
Other**	6	1	2	3
Total	26	5	11	10

*No studies used interrupted time series, cohort study or economic evaluation methodologies.

**The ‘Other’ category includes a description of an intervention with cases; a comparative study of two groups; a non-controlled before and after study; a review of theories; a controlled feasibility study; and a non-controlled before/after feasibility study.

The literature review highlighted heterogeneity in the definitions of ‘physical activity’. Studies identified and examined diverse possible activity interventions, including dance, exercises of various types and walking. None of the studies considered the physical activity aspects of activities of daily living such as housework and gardening. Participants were not always identified in terms of the types of dementia they might have: this is particularly problematic in the light of the various impacts considered, including cardio-vascular, cognitive, and well-being effects, which several studies suggested may vary according to type of dementia. As we will note, stage or degree of dementia was sometimes considered.

The three reviews included [22,23,25] all conclude that on balance, physical exercise has potential benefits for people with dementia. They all urge a degree of caution however, referring to the heterogeneity of the studies in terms of the wide range of interventions and outcomes considered, the tendency for small sample sizes, and gaps in the research.

In terms of identifying how physical activity may benefit people with dementia), the studies highlight benefits for cognition and mood (12 studies and two negative results), for behaviour (six studies) and for physical condition (eight studies) with three studies identifying potential benefits only. Only one study [26] explicitly considered sociability in relation to ease and positivity of interaction offered through dancing, seeing this as a clear benefit.

Some studies identify significant effects in terms of cognitive and executive function. Several of these focused on people with Mild Cognitive Impairment (MCI) or ‘early stage’ dementia. Buettner et al’s review [22], which included a consensus panel exercise, identified ‘potential’ for exercise to prevent and treat early stage AD. Others were more definite in their conclusions For example, Baker et al’s [27] RCT identifies that high intensity aerobic exercise, as compared with stretching exercise, can improve executive function for women with MCI. Burns et al. [28] find that cardiovascular fitness in people with early AD shows an association with reduced brain atrophy. Batman’s [29] work on aquatic exercise for people with mild-moderate AD suggested that higher levels of participation produced greater improvements in overall functioning, as compared with lower levels. The control group who did not participate showed decline in functioning.

Studies that specifically consider effects for ‘late stage’ dementia include Dayanim et al. [30], whose pre/post-test study identified significant decreases in problems with speech and recognition following an exercise programme. Francese et al. [31] found that a programme including hand grip and muscle strength exercises did increase muscle strength of people with ‘severe dementia’: they argue that increased muscle strength can support dignity, as people are better able to move from place to place and to use bathrooms more easily and independently. It is notable that the physical activity was seen to have a social benefit.

Edwards et al’s [32] study included people with ‘moderate to severe’ dementia and tested the impact of chair-based exercise on measures of depression and anxiety. Immediately following the sessions, these measures showed an improvement, which appeared to be sustained 12 weeks later. A study involving a walking programme for people with ‘moderate’ dementia [33] found no significant improvements in cognition: the authors noted however that many of the participants also had cardio-vascular disease, emphasising one of the important complications of studies in this area. Friedman and Tappen [34] found that a group walking programme did improve communication of people with AD more than a programme of conversation alone.

Heyn et al’s [23] meta analysis of results from 30 trials did suggest overall that physical activity could improve physical and cognitive fitness, finding larger effect sizes for physical fitness than for aspects of cognitive health.

Five main mechanisms of actions were posited for why physical activity might affect the progression or symptoms of dementia. These were mostly derived from animal experiments [6,35] and are outlined in Table 2. These mechanisms are not necessarily mutually exclusive.

**Table 2:** Mechanisms of action for physical activity and dementia

**Mechanism of action**	**Description**
Vascular	Exercise could restore cerebral hypoperfusion, the decrease of the perfusion of the blood into the brain
Neurochemical	Exercise increases endorphin and serotonin levels in the brain, which may in turn increase the functioning of the central nervous system and enhance cognitive performance
Cognitive reserve	Reduction in cognitive deficits is achieved by activating brain plasticity and enhancing synaptogenesis and neurogenesis
Stress	Physically active individuals have more positive emotional feelings, which reduce stress and lead to lower susceptibility to cognitive impairment
Functional	Facilitates acquisition of spatial learning and memory

The animal models did not articulate well how the mechanisms would ‘translate’ into impact on functional, behavioural or cognitive outcomes for people with dementia. We therefore looked for other plausible mechanisms of actions described in the studies. Few considered why physical activity might impact on the outcomes measured, although some such as grip strength and walking speed were more related to the effect of physical activity on general physical functioning rather than dementia. One study [36] used the Neurodevelopmental Sequencing Program (NDSP) theory which suggests that behaviour, movement and functional losses in people with dementia occur in approximately reverse order of original development.

**Figure 2:**
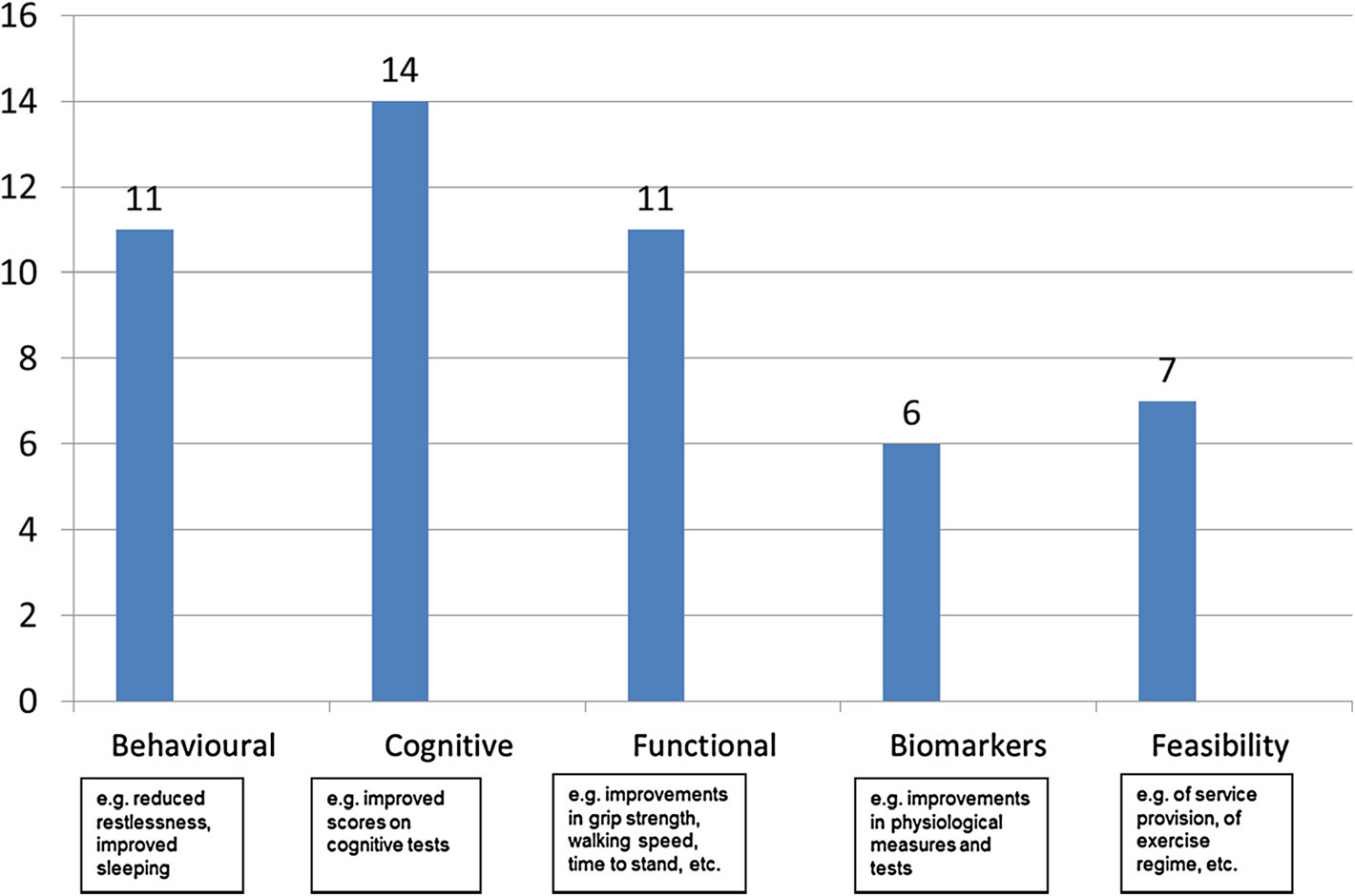
Outcomes considered in the 26 included studies.

Outcomes considered in the studies fell into five main categories–behavioural, cognitive, functional, biomarker, and feasibility (Figure 2). They broadly represented the researchers’ underlying hypotheses as to the causal chain, i.e. the link between undertaking physical activities and the effects they expected to see. The heterogeneity of the studies meant it was not possible to pool the results (and that was not the main purpose of the review). Rolland et al. [37] used a particularly wide range of outcomes: their study over 12 months found slower decline in ability to perform Activities of Daily Living (ADLs) for those who participated in the exercise programme, but no effects for behavioural, depression or nutritional status scores.

Studies additionally identified a range of possible benefits less directly related to the physical activity per se, including; group exercise as a means to unlock memories [38]; the potential to link physical exercise with beneficial cognitive exercise [34,39]; ways to augment the benefits of physical exercise such as through using supplements [40]; and the significance of social and emotional engagement that physical activity interventions often included [41,42].

The literature highlights that exercise interventions, some by design and some in their results, may link physical activity with associated activity, particularly social interactions and find social outcomes. Arakawa-Davies [38] for example links dance/movement therapy with reminiscence, and sees prompting reminiscence as a key result of the therapy. Holliman et al. [43], whose key outcome measure is behavioural change, found improvements in behaviour through delivery of a group physical activity programme: this study however raises questions about whether the engagement involved was more significant than the physical exercise per se. Palo-Bengtsson and Ekman [26] unusually focused on emotional responses as their key outcome, specifically identifying positive emotions such as joy and amusement: although this was a tiny study (with 6 participants), it highlights the potential range of outcomes that may follow, and explores the social and emotional dimensions of the interventions concerned, in this case social dancing and walks. Van den Winckel et al’s [42] intervention entailed exercise to music: in their view, the music was an essential component of the cognition improvements that were shown by participants. However, their sample was also small (15 in the intervention group and 10 controls), and whilst they raise interesting questions, these require further research.

Improving experiences in institutional care is a further concern. For example, Binder et al. [40] established that it was feasible to deliver a structured exercise programme to people with dementia living in residential care and were able to identify improved physical functioning with participation in the programme. Christofoletti et al. [39] found that physical therapy and exercise conferred physical benefits in terms of improved balance for care home residents, and limited impact on verbal fluency and executive function, but not on global cognition. Dorner et al’s [44] programme of strength and balance training also produced good results in terms of physical measures, but although the treatment group showed improvements in MMSE scores, these were not significantly different from those in the control group. Like Edwards [32] (above) Eggermont et al. [20] were concerned with psychological well being of people with dementia in nursing homes: they found that a hand movement programme affected mood positively.

Other settings are relatively neglected in the literature. Steinberg et al’s [21] study of an exercise programme delivered in people’s own homes suggested that whilst people’s physical performance improved, measures of depression and quality of life worsened. Despite establishing that exercise programmes could feasibly be delivered at home, they were unable to demonstrate wholly positive benefits, and argue for further research.

### Survey

Full survey results are given in the Additional file 1: Table S8. The largest groups of respondents were activities coordinators (24%), care home managers (24%), managers of other services (16%) and occupational therapists (14%). Most were female, reflecting the structure of the professions concerned. They were generally concerned with the design and delivery of activity programmes, suggesting first hand knowledge. Less than half had received training in delivering physical activities, much of which related to older people generally, and was not dementia specific. More than half the programmes described were provided in care homes, and under half in community settings, with a few in hospitals or elsewhere, reflecting the literature. There was a small majority of public over private and third sector provision and 60% of respondents were located in Scotland.

Most programmes were delivered by specialist activity coordinators (54%) with Allied Health Professionals (AHPs) and care workers or care assistants also involved, and 17% including volunteers. Participants in the activity programmes were reported to be in the older age groups and most programmes included a general population of older people, rather than being exclusively for people with dementia. Eligibility criteria were applied in one third of examples, relating either to existing use of other services or to the individual, such as being assessed as having potential to benefit.

The majority of participants (62%) were care home residents and care homes were the most usual locations involved. Only 5% of programmes were reported to be delivered in people’s own homes. Family caregivers could join the programme in 65% of cases. The average number of participants was 8.5, with a range of 1–60. On average, programmes had been running for four years, with the oldest having started in 1991. The predominant funders were local authorities and the NHS. A small number (14%) took fees from participants.

Many different physical activities were mentioned, the most frequent being seated exercise with music, walking, exercises, dancing and ball games such as bowling or skittles. Fourteen per cent of respondents mentioned ordinary ADLs, including housework and gardening as physical activity, reflecting the broad definition we had used in designing the research, and indicating that physical activity is not perceived only as specifically designed ‘exercise’ activities in the field (in contrast to the literature). Clearly, respondents recognised ADLs as opportunities to promote physical activity. Indicating the rationale for the programmes, respondents most commonly cited enjoyment (27%), social interaction (26%) and well-being (19%). Interestingly, these outcomes were not widely referred to in the literature. Forty two per cent of the programmes had been evaluated, though this had mostly been done internally (78%). The evaluations do not seem to have produced robust evidence of success (or otherwise), and the respondents made little comment on them.

### Interviews

The data provided real world examples of physical activity programmes, describing their development and current functioning. The programmes provided people with dementia with a wide range of activities including exercise programmes; walking groups; leisure club activities such as swimming and badminton; golf; gardening; dancing; computer games such as Wii; bowling, both indoor and outdoor; Tai Chi; and others. Activities often combined physical activity with social interaction to enhance the experience for people with dementia. Some programmes focused on activities considered particularly meaningful for participants such as gardening, games and dancing.

There were few examples of specific theory or thinking behind programmes; rather, interviewees talked about their programmes in terms of the outcomes and benefits for those taking part. One respondent did discuss theory, drawing on their occupational therapy knowledge to support normalization for those attending their programme that provided access for older people to a local leisure centre. Programmes often developed organically in response to a range of possible factors such as changes to client or resident populations; or the presence of an individual or group with a specific interest in physical activity who acted as champions. Programmes might be set up in response to recognition of a problem or to help find new ways to cope with changing clients. Sometimes multiple factors were involved.

There were several examples of individuals who acted as champions in delivering programmes. Due to the challenges of provision described below, their importance was clear. They could generate support within the service setting, secure funding and ensure the programme continued. However, when champions moved on, this could be problematic for the continuation of services.

Other programmes developed as a result of ideas spreading amongst organisations keen to spread best practice. Some successful projects had gone on to influence the development of new programmes in different areas or organisations:

Because it was so successful, I was given a job as an activity coordinator, and the idea is to develop this kind of philosophy throughout the city and that’s slowly and surely what we’re doing. (Interview 9103).

Participants shared several challenges that were overcome to set up the programmes. Unsurprisingly funding was the main issue raised; both a lack of it and a need to be innovative to secure it.

Many examples were given of innovative approaches to funding activity programmes including: fund raising with family and friends of clients; seeking donations of, for example, seeds and cuttings for a gardening group; accessing different types of grants such as those for healthy living or grants from external agencies or local businesses, and asking participants to contribute to costs by paying lunch money or for travel to and from the programme.

Then I found out that if we used that, [healthy eating] it sounds mercenary but it’s true. If we use that as a platform to get more money for funding, we’re more likely to get money and we did. (Interview 9103).

Activity programmes often required broad support within a service or organisation to ensure the continuation of projects. Individuals often had commitment to providing physical activity but without support from managers and the wider organisation any initiatives were unlikely to succeed.

As much as you may want to as a therapy department or as an individual OT want to get things going, then you do need to have some kind of commitment from the wider service, and that has been important…I think this particular scheme has worked because it goes across the services. (Interview 7969).

It is not always possible to recruit staff with the right levels of skills and knowledge for some physical activities. Many members of staff who provide physical activity programmes do so as part of their wider role at work and they may not be given protected time for these activities, which may not therefore have any priority.

Interviewees highlighted similar outcomes to the survey respondents, focusing on fun and enjoyment; well being and quality of life; and self esteem and confidence rather than specific changes in physical and cognitive abilities, although these were also highlighted by some. Several interviewees talked about the broad benefits provided, encompassing physical, social and emotional aspects.

The views seem to be quite similar, that people feel as if it’s assisting them physically, mentally and socially, promotes their health and wellbeing and kind of helps to maintain a lot of their abilities as well. (Interview 5596).

Examples given of physical benefits to participants included increased strength, better balance, increased mobility, better sleep patterns, reduction in falls and in one case a reduction in wandering behaviour. For example:

She’s developed a lot more strength and she now can get herself to the toilet and back. So that in itself is great. (Interview 9103).

Respondents reported that programmes provided social and emotional benefits to the participants. Participants were seen to socialise more with others during and after exercise and to draw pleasure and enjoyment from their participation. The improvements in mood could last long after the activity finished. Interviewees also mentioned the positive impact on self esteem and confidence seen among participants:

Perhaps from the point of view of feeling better about themselves, it’s a group of people who sometimes feel that they’re finished. You know, they can’t remember things, you know, they’re not very happy with themselves, and I think to find something that they can do [is positive]. (Interview 5692).

Programmes could also offer participants opportunities for meaningful activity through activities such as gardening, games and housework. These activities could also help people maintain or regain skills as well and promoting a normal environment for them:

I think it’s about maintaining skills as well, particularly with things like the gardening, which some of the men really enjoy. And they have a good workout sometimes… And it’s normality… and keeping the skills that they’ve got. (Interview 8682).

### Learning from the three evidence bases

The three complementary sets of evidence were intended to understand and explain the potential role of physical activity in reducing the progression and/or symptoms of dementia. The literature review identified a range of mechanisms and outcomes related to either the physical activity directly or indirectly through, for example the social engagement and unlocking memories [38]. However, the review also demonstrated limitations of the available evidence concerning the benefits or otherwise of physical activity for people with dementia.

The scientific literature identifies a multitude of potential interventions, delivered for a wide range of reasons. From the survey and the interviews, it is clear that many practitioners see physical activity as worthwhile to promote. However, a striking finding of the study concerns the lack of alignment between the scientific literature and the practice of service delivery. The scientific literature is heterogeneous, studying many different interventions, which are examined for evidence of delivery of a wide range of hypothesised benefits. In general, studies consider uni-dimensional outcomes (even if multiple) such as scores on particular tests, or physical measures such as grip strength or balance. Overall, the literature indicates a balance of evidence in favour of benefit, despite the focus on such a wide range of possibilities. Some studies consider the impact of physical activity on the disease course per se, and 12 find evidence of benefits for cognition and/or mood.

Our data suggest that practitioners by contrast look for different kinds of outcomes–they are particularly interested in well-being, sociability and enjoyment, and pay less attention to outcomes such as falls prevention, or measures of physical or cognitive improvement. Their perspectives are generally more holistic, and in discussing them they focus on meaning and purpose, and promoting self worth for people with dementia.

## Discussion

Service providers did not explicitly draw on scientific evidence, but rather on practical experience of benefits perceived. This was notably the case in care homes in which respondents considered the need to relieve monotony and find things to occupy residents. Despite not drawing explicitly on evidence, nevertheless the services provided did not seem to go against the available scientific evidence of likely benefit. There are few high quality RCTs in the area, and it could be concluded that more of these are needed. However, it is not clear whether this type of intervention is readily amenable to controlled trials. Physical activity interventions emerge as particularly complex and the evidence from the real world experience highlights significant difficulties in achieving the level of control that might be needed. In itself, this may explain why so few studies exist, but it also draws attention to the need to consider other kinds of evidence. Our review identified several studies which did not readily match Cochrane-type quality criteria, but nevertheless produced interesting and informative results. A particular area in which studies are missing is that of community dwelling older people with dementia: cost and logistical difficulties are highlighted as barriers, again suggesting the need to pursue other types of research design.

Relatedly, the practicalities of research in this area emerged as challenging. Many studies reported difficulties of recruitment to studies e.g. [29,32] and of sustaining participation e.g. [37,41,43]. Yu and Kolanowski [45] reported particular difficulty in involving primary care providers: this proved a significant barrier in the light of the role of these professionals as gatekeepers to the physical activity intervention. Several studies [20,33,36,43] highlighted the need to be sure that physical interventions were suitable for participants’ physical needs and abilities, which could vary significantly. This was linked with an issue relating to what types of physical activity might be appropriate for people at different stages in their dementia journey. The combined need to consider physical and cognitive impairment in designing interventions could lead to a study recruiting from a very restricted population of people with dementia, if a controlled study was envisaged. The issue of supporting people to engage in physical activity through appropriate stimulation and instruction was not generally explored in the literature, though it was discussed by service providers who referred for example to the need for staff or volunteers to do this.

Service providers suggested a need for ongoing resource-based support. Staffing is an issue for many, as a high staff:client ratio is required for some physical activity programmes. Providers of community-based services indicated that demand for their services outstrips their abilities to provide them. Care home providers generally indicate that there is no specific funding for activity programmes, limiting the range of physical activities offered, possibilities for staff training and future service development. Many providers raise funds to support service provision, and a number use or are considering using volunteers although this is not considered appropriate for all types of physical activity provision.

Service providers express information-based support needs, including needs for inter-provider knowledge exchange to disseminate best practice and share lessons learned and suggestions for novel and innovative activities and needs for information-based support around appropriate formal evaluation processes. Several highlighted the benefits to their programmes of working with other service providers, both to people with dementia and to other client groups. There is a need to help those developing physical activity services to understand the range of possibilities for cooperation and to help them identify and network with suitable partners.

## Conclusions

The scoping study reveals several gaps in the scientific understanding of physical activity for people with dementia and a notable lack of evidence base linked to theory for many interventions. There appears to be general consensus that physical activity is ‘beneficial’ for people with dementia and a wide range of benefits is both suggested in practice and somewhat supported in the literature. There is a general lack of clarity regarding how physical activity interventions work, what outcomes can be expected, and what outcomes are sought.

This is not to say that physical activity interventions need to await further scientific study–there are strong indications of benefits in terms of well-being and quality of life, and in terms of physical benefits such as improved balance (preventing falls), and grip strength (supporting independence in ADLs). Physical activity does not appear to be harmful per se, with the proviso that it needs to be appropriate to the individual. There is other evidence from practice experience of delivering services that improved quality of life is important for supporting people to live better with dementia: good quality of life, including sociability appears to maintain people at a higher level of functioning for longer, support informal carers and also deliver some physical benefits.

## Competing interests

The authors declare that they have no competing interests.

## Authors’ contributions

AB, RJ and LM jointly conceived the project. AD joined the team as Research Fellow. AD led the literature review with all authors reading papers; LM led the survey and interview elements of the research and wrote up the survey and interview findings. AB led the writing of the paper, with all other authors contributing sections and commenting on drafts. All authors read and approved the final manuscript.

## Pre-publication history

The pre-publication history for this paper can be accessed here:

http://www.biomedcentral.com/1471-2318/13/129/prepub

## Supplementary Material

Additional file 1 **Physical activity for people with dementia: a scoping study. Table S1.** Search terms used in the review. **Table S2.** Bibliographic databases searched. **Table S3.** Four point scoring system. **Table S4.** Scores on 4-point scale for remaining 216 abstracts. **Table S5.** Examination of full text items for inclusion in review. **Table S6.** Literature included in the review: study populations, interventions, comparisons and outcomes (PICO table). **Table S7.** Key conclusions of included items. **Table S8.** Survey results. Click here for file

## References

[B1] World Health Organisation Global recommendations on physical activity for health 2010 Geneva: World Health Organisation 26180873

[B2] Caspersen CJ Powell KE Christenson GM Physical activity, exercise, and physical fitness: definitions and distinctions for health-related research Public Health Rep 1985 100 126 131 3920711 PMC1424733

[B3] Brookmeyer R Evans DA Hebert L Langa KM Heeringa SG Plassman BL Kukull WA National estimates of the prevalence of Alzheimer’s disease in the United States Alzheimers Dement 2011 7 61 73 10.1016/j.jalz.2010.11.007 21255744 PMC3052294

[B4] Wilson RS Weir DR Leurgans SE Evans DA Hebert LE Langa KM Plassman BL Small BJ Bennett DA Sources of variability in estimates of the prevalence of Alzheimer’s disease in the United States Alzheimers Dement 2011 7 74 79 10.1016/j.jalz.2010.11.006 PMC3145367 21255745

[B5] Alzheimer’s Disease International (ADI) World Alzheimer report 2009 http://www.alz.co.uk/research/files/WorldAlzheimerReport.pdf

[B6] Kramer FK Erikson KI Capitalizing on cortical plasticity: influence of physical activity on cognition and brain function TRENDS Cogn Sci 2007 11 342 348 10.1016/j.tics.2007.06.009 17629545

[B7] Hokkanen L Rantala L Remes AM Harkonen B Viramo P Winblad I Dance and movement therapeutic methods in management of dementia: a randomized, controlled study J Am Geriatr Soc 2008 56 771 772 10.1111/j.1532-5415.2008.01611.x 18380687

[B8] Arcoverde C Deslandes A Rangel A Rangel A Pavao R Nigri F Engelhardt E Laks J Role of physical activity on the maintenance of cognition and activities of daily living in elderly with Alzheimer’s disease Arq Neuro-Psiquiat 2008 66 323 327 10.1590/S0004-282X2008000300007 18641864

[B9] Teri L Logsdon RG McCurry SM Exercise interventions for dementia and cognitive impairment: the Seattle Protocols J Nutr Health Aging 2008 12 391 394 10.1007/BF02982672 18548177 PMC2518041

[B10] Society A’s Dementia: out of the shadows 2008 London: Alzheimer's Society

[B11] Forbes D Forbes S Morgan DG Markle-Reid M Wood J Culum I Physical activity programs for persons with dementia Cochrane Database Syst Rev 2008 3 CD006489 10.1002/14651858.CD006489.pub2 18646158

[B12] Abraham C Michie S A taxonomy of behavior change techniques used in interventions Health Psychol 2008 27 379 387 18624603 10.1037/0278-6133.27.3.379

[B13] MRC Developing and evaluating complex interventions: new guidance 2008 http://www.mrc.ac.uk/Utilities/Documentrecord/index.htm?d=MRC004871

[B14] Pawson R Greenhalgh T Harvey G Walshe K Realist synthesis: an introduction 2004 University of Manchester: ESRC Research Methods Programme RMP Methods Paper 2/2004

[B15] Pawson R Greenhalgh T Harvey G Walshe K Realist review: a new method of systematic review designed for complex policy interventions J Health Serv Res Po 2005 10 Suppl 1 21 34 10.1258/1355819054308530 16053581

[B16] Moher D Liberati A Tetzlaff J Altman DG The PRISMA Group: Preferred reporting items for systematic reviews and meta-analyses: the PRISMA statement PLoS Med 2009 6 e1000097 10.1371/journal.pmed.1000097 19621072 PMC2707599

[B17] Centre for Reviews and Dissemination (CRD) Undertaking systematic reviews of research on effectiveness: CRD Report No. 4 2001 2

[B18] Cochrane Effective Practice and Organisation of Care (EPOC) group Data Extraction Form’, part of the EPOC Data Collection Template’. s 2002 http://epoc.cochrane.org/epoc-author-resources

[B19] Critical appraisal skills programmehttp://www.casp-uk.net/

[B20] Eggermont LHP Knolb DL Hol EM Swaab DF Scherder EJA Hand motor activity, cognition, mood, and the rest–activity rhythm in dementia A clustered RCT Behav Brain Res 2009 196 271 278 10.1016/j.bbr.2008.09.012 18926856

[B21] Steinberg M Leoutsakos J-MS Podewils LJ Lyketso CG Evaluation of a home-based exercise program in the treatment of Alzheimer’s disease: the maximizing independence in dementia (MIND) study Int J Geriatr Psych 2009 24 680 685 10.1002/gps.2175 PMC5172460 19089875

[B22] Buettner L Richeson NE Yu F Burgener SC Buckwalter KC Beattie E Bossen AL Fick DM Fitzsimmons S Kolanowski A Rose K Pringle Specht JK Testad I McKenzie SE Evidence supporting exercise interventions for persons in early-stage Alzheimer’s disease Am J Recreation Ther 2008 7 17 24 10.1007/BF02982159 PMC12892630 18165840

[B23] Heyn P Abreu BC Ottenbacher KJ The effects of exercise training on elderly persons with cognitive impairment and dementia: a meta-analysis Arch Phys Med Rehab 2004 85 1694 1704 10.1016/j.apmr.2004.03.019 15468033

[B24] Littbrand H Rosendahl E Lindelöf N Lundin-Olsson L Gustafson Y Nyberg L A high-intensity functional weight-bearing exercise program for older people dependent in activities of daily living and living in residential care facilities: evaluation of the applicability with focus on cognitive function Phys Ther 2006 86 489 498 16579666

[B25] Scherder E Eggermont L Sergeant J Boersma F Physical activity and cognition in Alzheimer’s disease: relationship to vascular risk factors, executive functions and gait Rev Neuroscience 2007 18 149 158 10.1515/revneuro.2007.18.2.149 17593877

[B26] Palo-Bengtsson L Ekman S Emotional response to social dancing and walks in persons with dementia Am J Alzheimer’s Dis 2002 17 149 153 10.1177/153331750201700308 PMC10833923 12083344

[B27] Baker LD Frank LL Foster-Schubert K Green PS Wilkinson CW McTiernan A Plymate SR Fishel MA Stennis Watson G Cholerton BA Duncan GE Mehta PD Craft S Effects of aerobic exercise on mild cognitive impairment Arch Neurol Chicago 2010 67 71 79 10.1001/archneurol.2009.307 20065132 PMC3056436

[B28] Burns JM Cronk BB Anderson HS Donnelly JE Thomas GP Harsha A Brooks WM Swerdlow RH Cardiorespiratory fitness and brain atrophy in early Alzheimer’s disease Neurology 2008 71 210 216 10.1212/01.wnl.0000317094.86209.cb 18625967 PMC2657657

[B29] Batman MW The effects of therapeutic aquatic exercise on patients with Alzheimer’s disease, PhD thesis 1999 Cincinnati, Ohio: The Graduate School of The Union Institute

[B30] Dayanim S The acute effects of a specialized movement program on the verbal abilities of patients with late-stage dementia Alzheimer’s Care Today 2009 10 93 98

[B31] Francese T Sorrell J Butler FR The effects of regular exercise on muscle strength and functional abilities of late stage Alzheimer’s residents Am J Alzheimer’s Dis 1997 12 122 127 10.1177/153331759701200305

[B32] Edwards N Gardiner M Ritchie DM Baldwin K Sands L Effect of exercise on negative affect in residents in special care units with moderate to severe dementia Alz Dis and Assoc Dis 2008 22 362 368 10.1097/WAD.0b013e31818ecbbc 18978600

[B33] Eggermont LHP Swaab DF Hol EM Scherder EJA Walking the line: a randomised trial on the effects of a short term walking programme on cognition in dementia J Neurol Neurosurg Psychiatry 2009 80 802 804 10.1136/jnnp.2008.158444 19531688

[B34] Friedman R Tappen RM The effect of planned walking on communication in Alzheimer’s disease J Am Geriatr Soc 1991 39 650 654 2061529 10.1111/j.1532-5415.1991.tb03617.x

[B35] Cotman CW Berchtold NC Physical activity and the maintenance of cognition: learning from animal models Alzheimers Dement 2007 3 S30 S37 10.1016/j.jalz.2007.01.013 19595972

[B36] Buettner LL Fitzsimmons S Recreational therapy exercise on the special care unit: impact on behaviours Am J Recreation Ther 2004 3 8 24

[B37] Rolland Y Pillard F Klapouszczak A Reynish E Thomas D Andrieu S Riviere D Vellas B Exercise program for nursing home residents with Alzheimer’s disease: a 1-year randomized, controlled trial J Am Geriatr Soc 2007 55 158 165 10.1111/j.1532-5415.2007.01035.x 17302650

[B38] Arakawa-Davies K Dance/movement therapy and reminiscence: a new approach to senile dementia in Japan Arts Psychother 1997 24 291 298 10.1016/S0197-4556(97)00031-2

[B39] Christofoletti G Oliani MM Gobbi S Stella F Gobbi LTB Canineu PR A controlled clinical trial on the effects of motor intervention on balance and cognition in institutionalized elderly patients with dementia Clin Rehabil 2008 22 618 626 10.1177/0269215507086239 18586813

[B40] Binder EF Implementing a structured exercise program for frail nursing home residents with dementia: issues and challenges J Aging Phys Activ 1995 1995 3 383 395

[B41] Hernandez SSS Coelho FGM Gobbi S Stella F Effects of physical activity on cognitive functions, balance and risk of falls in elderly patients with Alzheimer’s dementia Rev Bras Fisioter 2010 14 68 74 10.1590/S1413-35552010000100011 20414564

[B42] Van de Winckel A Feys H De Weerdt W Dom R Cognitive and behavioural effects of music-based exercises in patients with dementia Clin Rehabil 2004 18 253 260 10.1191/0269215504cr750oa 15137556

[B43] Holliman DC Orgassa UC Forney JP Developing an interactive physical activity group in a geriatric psychiatry facility Activities Adapt Aging 2001 26 57 69 10.1300/J016v26n01_04

[B44] Dorner T Kranz A Zettl-Wiedner K Ludwig C Rieder A Gisinger C The effect of structured strength and balance training on cognitive function in frail, cognitive impaired elderly long-term care residents Aging Clin Exp Res 2007 19 400 405 10.1007/BF03324721 18007119

[B45] Yu F Kolanowski A Facilitating aerobic exercise training in older adults with Alzheimer’s disease Geriatr Nurs 2009 30 250 259 10.1016/j.gerinurse.2008.11.001 19665667

