# Supplementary material for: Physical activity for people with dementia: a scoping study
Source: BMC Geriatr. 2013 Nov 26;13:129. doi: 10.1186/1471-2318-13-129 (PMC4222572; doi:10.1186/1471-2318-13-129)
Supplement: Additional file 1 — Physical activity for people with dementia: a scoping study. Table S1. Search terms used in the review. Table S2. Bibliographic databases searched. Table S3. Four point scoring system. Table S4. Scores on 4-point scale for remaining 216 abstracts. Table S5. Examination of full text items for inclusion in review. Table S6. Literature included in the review: study populations, interventions, comparisons and outcomes (PICO table). Table S7. Key conclusions of included items. Table S8. Survey results. [file 1471-2318-13-129-S1.docx]

**Physical activity for people with dementia: a scoping study**

**Supplementary tables**

***Table 1 Search terms used in the review***

| (dement* or Alzheimer* or (Lewy* bod*) or (cognit* impair*)) and exercis*  (dement* or Alzheimer* or (Lewy* bod*) or (cognit* impair*)) and (physical activit*)  (dement* or Alzheimer* or (Lewy* bod*) or (cognit* impair*)) and swim*  (dement* or Alzheimer* or (Lewy* bod*) or (cognit* impair*)) and gym*  (dement* or Alzheimer* or (Lewy* bod*) or (cognit* impair*)) and walk*  (dement* or Alzheimer* or (Lewy* bod*) or (cognit* impair*)) and danc*  (dement* or Alzheimer* or (Lewy* bod*) or (cognit* impair*)) and yoga  (dement* or Alzheimer* or (Lewy* bod*) or (cognit* impair*)) and ((tai chi) or (tai ji))  (dement* or Alzheimer* or (Lewy* bod*) or (cognit* impair*)) and stretch*  (dement* or Alzheimer* or (Lewy* bod*) or (cognit* impair*)) and sport*  (dement* or Alzheimer* or (Lewy* bod*) or (cognit* impair*)) and (physical train*) |
| --- |

***Table 2: Bibliographic databases searched***

| **Bibliographic database** |
| --- |
| BIOSIS Previews (via Web of Knowledge) |
| CINAHL |
| CSA Illumina (ASSIA + Social Services Abstracts + Sociological Abstracts databases) |
| IngentaConnect |
| OVID (PsychINFO + Medline + British Nursing Index + Embase + Social Work Abstracts databases) |
| Web of Science (via Web of Knowledge) |

***Table 3. Four-point scoring system***

| Scoring System  Score 1 point for a positive answer to each of the following questions, to provide overall score for article between 0 and 4:  1. Does the subject matter of this article or the research on which this article is based specifically include people with dementia or cognitive impairment?  2. Does this article suggest or explain mechanisms of action for benefitting from physical activity (physiological, psychological or social)?  3. Does this article describe or evaluate a specific form of physical activity, rather than referring to physical activity in general?  4. Does this article identify a specific research study or review a collection of studies?  Record scores  Keep note of any low scoring articles which you feel deserve full text examination |
| --- |

***Table 4. Scores on 4-point scale for remaining 216 abstracts.***

| **Score** | **Frequency** | **Cumulative frequency** |
| --- | --- | --- |
| 4 | 12 | 12 |
| 3.5 | 20 | 32 |
| 3 | 68 | 100 |
| 2.5 | 42 | 142 |
| Less than 2.5 | 74 | 216 |

***Table 5. Examination of full text items for inclusion in review***

| **Score on 4-point scale** | **Number of articles** | **Number obtained (reason item(s) not obtained** | **Number fully evaluated** | **Reasons for no full evaluation** |
| --- | --- | --- | --- | --- |
| 4 | 12 | 10 (PhD thesis, not available online; ILL requested but not delivered) | 6 | - Only abstract in English - full text in Chinese - Text of item details study protocol not study itself - Item is a study of a multimodal intervention with no separation of effects of physical activity - Item is a study primarily about effects of light exposure, physical activity is incidental |
| 3.5 | 20 | 19 (PhD Thesis, not available online) | 12 | - Item is a systematic review of psychosocial interventions, half of which have no physical activity component - Item is a short review of another article - Item is a study of a sensorimotor neurodevelopmental sequencing programme – not all programme activities are physical activity and there is no separation of the physical activity component - Item describes general conclusions of a consensus report – specific review of physical activity interventions is described elsewhere and that article has been fully evaluated in the review - Item repeats description of a study reported elsewhere and fully evaluated in review - Item does not describe a specific physical activity intervention - Item is a review of principles behind protocols – studies mentioned were multimodal interventions without separate assessment of physical activity component |

***Table 6: Literature included in the review: study populations, interventions, comparisons and outcomes (PICO table)***

| **Author(s) and date of publication** | **Study type** | **Study population (sample size)** | **Intervention** | **Comparison** | **Outcomes of interest (outcome measure)** | **Assessed quality of study** |
| --- | --- | --- | --- | --- | --- | --- |
| Arakawa-Davies (1997) | Description of intervention plus case examples | Temporarily hospitalized senile dementia patients in a psychiatric facility in a large city in Japan with diagnoses of organic dementia or Alzheimer's Disease.  (n=12) | Dance / movement therapy session (DMT), incorporating introductions, warm-up, theme development and closing / cool-down.  60 minute sessions, once per week, for 12 weeks. | No comparison | Successful reminiscence (observation of degree of active participation in movements and attendant discussions) | Low |
| Baker et al (2010) | Randomised Controlled Trial (RCT) | Older adults living in the community and diagnosed with amnestic MCI (single or multiple domain) using Petersen criteria, self-reporting as sedentary and meeting health and medicines related inclusion criteria.  (n=33) | Aerobic exercise at 75% to 85% of HR reserve using a treadmill, stationary bicycle, or elliptical trainer (study indicates treadmill was most commonly chosen apparatus).  60-minute sessions, 4 times per week, with 6-week build-up then 20 weeks at target heart rates. | Prescribed routine of stretching and balance exercises, maintaining HR at or below 50% of HR reserve.  60-minute sessions, 4 times per week, for 26 weeks. | Executive function (Symbol-Digit Modalities, Verbal Fluency, Stroop, Trails B, and Task Switching testing).  Memory (Story Recall, List Learning, and Delayed-Match-To-Sample)  Glucoregulation and insulin sensitivity (hyperinsulinemic-euglycemic clamp method), peak cardiorespiratory capacity (graded exercise treadmill test), percentage body fat (dual energy  x-ray absorptiometry), BMI.  Total cholesterol levels, low-density lipoprotein levels, high-density lipoprotein levels, triglyceride levels, cortisol levels, total plasma BDNF levels adjusted for the contribution of activated platelets, levels of plasma IGF-I and mean levels of plasma Beta-amyloids 40 and 42 (radioimmunoassay). | Medium |
| Batman (1999) | Controlled Before and After Study (CBA) | Adults aged between 66 and 98 with diagnoses of mild to moderate Alzheimer's Disease attending an adult day care centre.  (n=24=total population) | Structured aquatic exercise in a heated indoor swimming pool aimed at increasing functional abilities and including gait training, range of motion, hip and knee flexion, abduction /adduction, upper extremity range of motion and balance. SOME subjects participated in other activities, such as aquacise, playing ball and (men) pull-ups and dips. 45-60 minute sessions, 2 times per week, for 18 weeks. | Non-exercising group | Behavioural symptoms (25-item behavioural scale in use at the day centre (Keeps you up at night; cries easily; becomes restless or agitated; becomes angry or irritable; swears or uses foul language; threatens people; steals or hides objects; wanders; aware of date; aware of time; aware of place; sleeps during day; able to feed self; goes to bathroom independently; is constipated; falls down; increase in appetite; decrease in appetite; more physical stamina; better balance; gets up and down easier; walks better; less awkward; requires less psychotropic medication; requires more psychotropic medication) assessed by observation and rated on a 4-point scale from 'Never' to 'Always'). | Low |
| Binder (1995) | Controlled Clinical Trial (CCT) | Residents of a nursing home with documented chronic cognitive impairment or a diagnosis of dementia and having difficulty with transfers or ambulation or a recent history of falls. Inclusion criteria: ability to ambulate 25 feet or more without assistance; no severe visual or auditory impairment; ability to follow simple verbal commands; no current participation in a skilled physical therapy program; current use of vitamin D in doses no greater than 2,800 IU per week; and no history of hypercalcemia, nephrolithiasis, or hyperparathyroidism.  (n=34) | Group exercise program incorporating 5-10 minutes of warm-up and cool-down exercises plus repetitions of a series of exercises designed to improve muscle strength, flexibility and leg speed performed to music using basic equipment.  60-minute sessions, 3 times per week, for 8 weeks.  In addition the intervention group received a bolus dose of 100,000 U vitamin D orally, then weekly supplements of 50,000 U and oral calcium carbonate, at doses of 1,000 mg/day. | Group exercise program as per intervention with same session length, frequency and study period. No vitamin D supplement, but oral calcium carbonate, at doses of 1,000 mg/day for 8 weeks. | Muscle strength (single and multiple times to stand; knee extensor torque at different angular movement speeds; 1 repetition maximum measure of hip extension).  Gait (time to walk 24ft, number of steps to walk 24ft).  Static balance (three variations on Romberg manoeuvre).  Serum calcium, phosphorous, and 25-hydroxy vitamin D (25(OH)D) levels | Medium |
| Buettner and Fitzsimmons (2004) | Controlled Before and After Study (CBA) | Residents in long-term care facilities in southwest Florida, aged 65 years+ with an existing diagnosis of dementia, stable on current medication, having MMSE of 24 or less, and having been identified by staff as having predominantly passive behaviours during mornings and agitated behaviours in the afternoons.  (n=20) | A choreographed exercise routine performed to music chosen by the participants, beginning with 'sensory experience' and including activities to promote range of motion, strength, and endurance using basic equipment such as a wooden dowel, resistance bands, ribbons, balloons, a ball, and free weights. 30-minute sessions, 7 mornings per week starting at 10am, for 4 weeks. | A choreographed exercise routine which mirrored the intervention in terms of content, intensity and frequency, but with sessions scheduled for the afternoon, 2.30pm start time. | Timing of intervention - morning or afternoon.  Agitation (Cohen-Mansfield Agitation Inventory (CMAI)) and passivity (Passivity in Dementia Scale (PDS).  Right and left grip strength (dynamometer), flexibility (Sit and Reach test, modified for wheelchair users). | Low |
| Buettner et al (2008) | Literature Review | Review includes 11 articles testing the effects of exercise interventions on a variety of outcomes for persons in early stage Alzheimer's disease (defined as having MMSE score of 18+, CDR score of 2.0 or less, or GDS score of 2 or less). | Articles are divided into 'multimodal interventions including exercise' and 'exercise only' interventions. | Various | Reviewed studies considered various outcomes, including improvements in cognition, physical and functional ability, depression and behavioural symptoms. | High |
| Burns et al (2008) | Comparative study of two groups | Older adults who met cognitive, mental and physical health and medicines use related inclusion criteria.  A non-demented group (n=64) and a group with early Alzheimer's Disease (n=57). | Symptom-limited graded treadmill test. | Inter-group comparison. | Levels of habitual physical activity (Physical Activity Scale in the Elderly (PASE)). Current respiratory fitness measured as maximum oxygen consumption, VO2(peak) (analysis of expired air), peak heart rate, exercise duration, and rating of perceived exertion (Borg Rating of Perceived Exertion scale).  Whole brain volume, white matter volume and gray matter volume (Structural Magnetic Resonance Imaging) | Medium |
| Christofoletti et al (2008) | Randomised Controlled Trial (RCT) | Medically fit older residents of a long-term psychiatric institution with diagnoses of dementias, no other neurological diagnosis or neuropsychiatric conditions associated to cognitive impairment, no prescriptions of antidepressant medications with central anticholinergic or sedation actions, and no drug-related impairment of cognition or balance.  (n=54) | Interdisciplinary programme of physiotherapy (kinesiotherapeutic exercises that stimulated strength, balance and cognition), occupational therapy (in-group arts and crafts activities) and physical education (in-group walking and exercises to stimulate strength, balance, motor coordination, agility, flexibility and aerobic endurance). 120-minute sessions, 5 times per week, for 26 weeks. | Two comparison groups:  Physiotherapy group received same kinesiotherapeutic exercises as used with intervention group but no occupational therapy or physical education. 60-minute sessions, 3 times per week, for 26 weeks.  Control group received no motor interventions. | Cognitive functioning (Brief Cognitive Screening Battery (BCSB), Semantic Verbal Fluency Test, Clock Drawing Test).  Physical functioning (14-item Berg Balance Scale, Timed Get-Up-and-Go Test). | Medium-High |
| Dayanim (2009) | Controlled Before and After Study (CBA) | Residents of the locked dementia unit in a hospital with diagnoses of stage 6 or 7 senile dementia or Alzheimer’s disease.  (n=22) | A movement therapy session for up to six participants plus group leader, incorporating initial stretching plus warm-up and cool-down movements for all body parts using self-touch, tossing / hitting balloons, playing catch with a ball or balloon filled with beads, rolling/kicking a large ball across floor, use of a co-oper band held by each of the participants in circle, and repeat balloon tossing.  20-minute sessions with pre-and post-session testing. Sessions repeated a maximum of 10 times. Frequency and total study period not specified. | No movement. Participants seated in common area were tested twice with an interval of 20 minutes between tests. Procedure was repeated a maximum of 3 times. Session frequency and total study period not specified. | Ability to recognise and name the colour of a first object and the identity of a second object (study-specific 2-question test battery). | Low |
| Dorner et al (2007) | Controlled Clinical Trial (CCT) | Residents of a geriatric long-term care facility with diagnoses of dementia, MMSE scores of greater than 10 and the ability to walk 5 metres or more with or without walking aids who met acute condition health-related study inclusion criteria. | Group training sessions instructed by a sports scientist and including warm-up, strength and balance training consisting of repetitions of exercises using basic equipment such as elastic resistance bands (therabands), soft weights, exercise balls, balance discs and small blocks, and cool down.  50-minute sessions, 3 times per week, for 10 weeks. | No training intervention. | Cognitive function (MMSE, German version).  Depression (Geriatric Depression Scale (GDS)).  Activities of daily living (Barthel-Index and functional independence measure (FIM).  Mobility (Tinetti test).  Muscle function (rated by a physiotherapist using study-specific assessment instrument).  BMI (height and weight, with lean body mass measured by bioelectric impedance analysis). | Medium |
| Edwards et al (2008) | Non- controlled before and after study | Residents of secured dementia-specific units in two long-term care settings with moderate to severe dementia (mean MMSE score of 11.6 (SD=5.9)), able to follow verbal commands and/or respond to verbal or visual cueing.  (n=36) | Chair-based exercise sessions consisting of various stretches and exercises using a 1lb weight, plus toe taps, leg thrusts, hamstring stretch, and walking if possible.  30-minute sessions, 3 times per week, for 12 weeks. | Before and after intervention. | 'Affect' (observer -rated anger, anxiety, and depression components from the Philadelphia Geriatric Center Apparent Affect Rating scale).  Cognitive status (MMSE).  Functional status (modified Katz Index of Independence in Activities of Daily Living (Katz ADL), yes/no response replaced by 1-5 rating scale for activities).  Participation (observation and rating on scale of 1-4) and compliance (actual exercise days/possible exercise days). | Low-medium |
| Eggermont et al (2009a) | Randomised Controlled Trial (RCT) | Nursing home residents a minimum of 70 years old with diagnoses of dementia and no apparent disability in hand motor function.  (n=61) | A program of hand movements performed in groups and based on movements especially designed for the population, including finger movements, pinching a soft ball, or handling a rubber ring, among others.  30-minute sessions, 5 times per week, for 6 weeks. | Structured group conversation to control for social aspects of group activity, comprising reading from selected texts recommended for specific population followed by casual conversation.  30-minute sessions, 5 times per week, for 6 weeks. | Face recognition and picture recognition (Rivermead Behavioural Memory Test).  Executive function (Digit span subtest from Wechsler Memory Scale-Revised, category fluency, stop signal task, and attention network test).  Mood (Geriatric Depression Scale (GDS), Dutch version).  Rest-activity, data collected using wrist-worn activity monitors (Interdaily stability, intradaily variability, and relative amplitude) | High |
| Eggermont et al (2009b) | Randomised Controlled Trial (RCT) | Residents across 23 care homes who were 70 years or older, had diagnoses of dementia and MMSE scores between 10 and 24, were able to walk for short distances with or without a walking aid, and had no visual disturbances, hearing difficulties, history of alcoholism, personality disorders, cerebral trauma, hydrocephalus, neoplasm or disturbances of consciousness.  (n=97) | Walked at a self-selected speed, accompanied by a psychology student, primarily on the wards or in public places in the care home.  30-minute sessions, 5 times peer week, for 6 weeks.  Missed interventions were subsequently caught up over the weekend. | 30-minute social visits from psychology students with the same frequency and during the same period as the intervention. | Face recognition and picture recognition (Rivermead Behavioural Memory Test).  List learning and recall (eight words test, measuring encoding, immediate recall, delayed recall, recognition).  Digit span (Wechsler Memory Scale-Revised).  Category fluency and letter fluency. | Medium |
| Francese, Sorrell and Butler (1997) | Randomised Controlled Trial (RCT) | Non-ambulatory residents of a nursing facility with diagnoses of late-stage Alzheimer disease who understood English language, were medically fit to participate, and required the assistance of one or two care caregivers to transfer.  (n=11) | An exercise session incorporating the use of music, games (for hand grips), beanbags, beach balls, squoosh ball, Velcro ball and mitt, as well as parachute leg weights.  20-minute sessions, 3 times per week, for 7 weeks. | Watching a music video encouraging viewers to sing along, followed by a snack.  20-minute sessions, 3 times per week, for 7 weeks. | Muscle strength in upper and lower extremities (physiotherapy assessment).  Balance (Tinetti test).  Ability to carry out activities of daily living (Changes in Advanced Dementia Scale (CADS)). | Low |
| Friedman and Tappen (1991) | Controlled Clinical Trial (CCT) | US-born Caucasian residents of two nursing homes with similar staff-patient ratios and treatment programs with diagnoses of probable Alzheimer's disease and moderate to severe cognitive impairment (MMSE score <19), no history of mental illness, mental retardation, stroke or head injury, no regular prescription of psychotropic medicines or current delusions or hallucinations, and assessed by their physician as being capable of participating in the walking program.  (n=30) | Planned walking in the grounds of the institution at the pace of the participant while engaging in conversation with the investigator.  30-minute sessions, 3 times per week, for 10 weeks. | Conversation only with the investigator.  30-minute sessions, 3 times per week, for 10 weeks. | Communication performance (Communication Observation Scale for the Cognitively Impaired (COS), a modification of the Social Reaction Rating Scale, and Communication Assessment for the Cognitively Impaired Scale (CAS)). | Medium |
| Hernandez et al (2010) | Controlled Clinical Trial (CCT) | Community-dwelling elderly people with Alzheimer’s disease living in an urban environment and with access to caregivers. | A supervised program of regular physical activity performed at 60-80% of maximum heart rate in groups with the help of trainees, structured to promote motor and cognitive stimulation of the participants both simultaneously or separately and develop coordination, agility, balance, flexibility, strength and aerobic capacity, and including stretching activities, weight training, circuits, pre-sport games, dance sequences, recreational activities and relaxation.  60-minute sessions, 3 times per week on non-consecutive days, for 26 weeks. | Normal routine (no exercise intervention). | Cognition (MMSE).  Balance, speed and agility (Berg Balance Scale (BBS),  Timed Up-and-Go test (TUG), and agility/dynamic balance test (AGIBAL) of the American Alliance for Health, Physical Education, Recreation and Dance (AAHPERD) test battery). | Low |
| Heyn, Abreu and Ottenbacher (2004) | Literature review | 30 trials included in the meta-analysis, generating sample size of 2020 subjects, 1023 in treatment groups and 997 in comparison groups. 72% women, 28% men, mean age 80, sd 6.1Years | Literature inclusion criterion: 'any exercise program or form of rehabilitative exercises, physical activity, fitness, or recreational therapy’. Many mixed modalities interventions. 17 studies based on walking or walking plus isotonic exercises, 3 on chair exercises, 3 on aerobic dance class, 2 on strength training with weights.  Variable length and frequency of intervention and duration of study. 20-150 minute sessions (mean 45), 1-6 times per week (mean 3.6), for 2-112 weeks (mean 23). | Various | Studies had to include at least one dependent variable from one of the categories: health-related physical fitness (cardiovascular, strength, flexibility, BMI), functional, cognitive, and behavioural outcomes. | High |
| Holliman, Orgassa and Forney (2001) | Randomised Controlled Trial (RCT) | Residents of a geriatric psychiatric facility with primary diagnoses of dementia, committed or sent to the facility by order of court because they were adjudged to be a danger to themselves or others, resident for at least three weeks, not participating in any other research study at the time of intervention and not scheduled to be transferred before the end of the study.  (n=14) | Group physical activity sessions designed to encourage interaction and socialisation and train gross and fine motor skills, including passing beanbags, balls or small sacks of marbles round the circle, balloon volleyball, kickball, tossing the ball of yarn around to create a giant spider web, and clapping hands, and finishing with a snack and further socialisation.  30-minute sessions, 3 times per week, for 2 weeks. | Not explicitly stated – assumed to be no intervention. | Cognitive function (MMSE).  Incidence of ‘Problem’ behaviours (behavioural subscale of the psychogeriatric dependency rating scale (PGDRS), plus patient behaviour rating sheet (PBRS) during interventions). | Medium |
| Lange-Asschenfeldt and Kojda(2008) | Review of theories regarding mechanisms of action | Focused on people in mid life at risk of cognitive decline, but also considers impact of physical activity on cognitive decline. | Review refers to physical activity generally, especially that affecting cardio-vascular health, rather than specific exercise / activities. | Not applicable | Interconnection between AD and VRFs and the impact of cerebrovascular and endothelial dysfunction on AD pathophysiology.  Molecular mechanisms of the beneficial effects of exercise on the vasculature such as activation of the vascular nitric oxide (NO) / endothelial NO synthase (eNOS) pathway, upregulation of antioxidant enzymes, and angiogenesis. | Medium |
| Littbrand et al (2006) | Controlled feasibility study | Older care home residents who were dependent in personal activities of daily living (dependent on assistance in one or more personal ADL according to the Katz index), able to stand up from a chair with armrests with help from no more than one person, and were cleared for participation from their physician.  (n=91, 47 with diagnoses of dementia) | Individually tailored physical activity sessions based on the High-Intensity Functional Exercise Program (the HIFE Program) developed for the Frail Older People–Activity and Nutrition Study in Umeå (FOPANU) study and consisting of a selection from 41 repetition-based exercises performed in functional weight-bearing positions and at a high intensity, if possible.  45-minute sessions, an average of 2.5 sessions per week, for 13 weeks. | ‘Social activity’ control group – no activity details provided. | Attendance.  Intensity of lower-limb strength and balance exercises in terms of weight and maximum number of repetitions.  Occurrence and seriousness of adverse events. | High |
| Palo-Bengtsson and Ekman (2002) | Qualitative research | Long-term attendees of a day care program at a care home with scores on the Gottries-Brane-Steen (GBS) scale fitting the criteria for dementia.  (n=6, 5 care home residents, one person living in the community) | 1) Monthly dance events in a large entertainment hall for residents, their relatives and caregivers, with a local band playing popular dance music and seating and tables arranged around a dance space.  2) Organised daily walks in small groups of people with dementia and their caregivers (some participants transported in wheelchairs).  45-minute interventions, once per month. | No comparator group / activity. | Emotional response to activities (analysis of video recordings of participants engaged in dancing and walking activities, adopting a phenomenonological approach and making inductive judgements based on facial expressions, bodily movements, body posture, eye contact, touch, and tone of voice). | Low |
| Rolland et al (2007) | Randomised Controlled Trial (RCT) | Residents of five care homes with at least 2 months’ residency and having diagnoses of probable or possible AD, no evidence of vascular dementia or Parkinson’s disease, no cardiac conditions that might deteriorate during exercise, and no diagnoses of a terminal illness with life expectancy of less than 6 months, and with the ability to transfer from a chair and walk at least 6 meters without human assistance. | An exercise program that included aerobic, strength, flexibility, and balance training. After warm-up stretching at least half of each session was walking round a circular trail created inside the nursing home, with walking interspersed with personalised strength, flexibility, and balance training at predetermined stations along the trail where guardrails in the corridor or foam rubber ground sheets could be used for safety.  60-minute sessions, 2 times per week, for 52 weeks. | Routine medical care. This group had no exercise or specific behaviour management training. The study design stipulated no  restriction in nursing, physiotherapy, medical care, advice, or any other healthcare support. | Ability to perform activities of daily living (Katz ADL).  Physical performance (6-meter walking speed, get-up-and-go test, and one-leg balance test).  Nutritional status (body weight and Mini-Nutritional Assessment (MNA)).  Behavioural disturbance (Neuropsychiatric Inventory (NPI)).  Depression (Montgomery-Asberg Depression Rating Scale (MADRS)). | Medium-High |
| Scherder et al (2007) | Literature review | Inclusion criteria for literature review included that studies include participants with diagnoses of Alzheimer's disease. | Literature review inclusion criterion: Only studies with programmes exclusively focused on exercise, physical activity or fitness training with NO other form of stimulation, e.g. talking | Various. | Literature review inclusion criterion: only studies where dependent variable was cognition. | Medium-Low |
| Steinberg et al (2009) | Randomised Controlled Trial (RCT) | Ambulatory older people with diagnoses of probable Alzheimer’s disease (MMSE >=10) living in the community but not in assisted living, having a stable medical history and general health and having a caregiver who spent at least 10 hours per week with them.  (n=27) | An exercise program with three components: (1) brisk walking or, no more than one day each week, some other comparable moderate-intensity aerobic activity; (2) strength training exercises using resistive bands and ankle weights; (3) Balance and flexibility training exercises incorporated shifting centre of gravity, tandem walks, forward and backward walks, and chair sit to stands.  Daily sessions of unspecified length, for 12 weeks. | A home safety  Assessment with two subsequent home visits reviewing the identified hazards, recommending interventions, and evaluating implementation. (Activities ensured that patient contact time was equivalent across groups and optimized masking of the research assistant who was rating the outcomes). | Functional performance (Yale Physical Activity Survey (YPAS), Timed 8-foot walk, Jebsen Total Time (JTT), which evaluates the total time taken to perform a range of hand movements associated with activities of daily living, Chair sit to stand test.  Cognitive functioning (cognitive battery incorporating Mini-Mental State Exam (MMSE), Boston Naming Test (BNT) and Hopkins Verbal Learning Test (HVLT).  Neuropsychiatric symptoms (Alzheimer’s Disease Quality Related Life Scale (ADQRL) and Neuropsychiatric Inventory (NPI), and Cornell Scale for Depression in Dementia (CSDD)  Caregiver burden (Screen for Caregiver Burden (SCB)). | High |
| Van de Winckel et al (2004) | Randomised Controlled Trial (RCT) | Female permanent residents at a psychiatric hospital with diagnoses of multiple infarct dementia or possible/probable Alzheimer's disease, MMSE scores of <24, responsive and with abilities to remain seated for 30 minutes, to respond to verbal or visual commands, to be able to mimic the movements of the therapist and to hear the music.  (n=25) | A group based exercise programme in a separate room away from other residents, in which participants sat in a circle facing the therapist and followed seated dance exercises focused on upper and lower body strengthening, as well as balance, trunk movements and flexibility training, performed to folkloric accordion songs, such as polka, folk, country and western music.  30-minute sessions, 7 times per week, for 13 weeks. | Daily individual one-to-one conversations  with the therapist in the ‘day room’, with the therapist taking care that  this conversation did not concern issues addressed by the MMSE. No music was played and patients were not asked to perform any movements.  30-minute sessions, 7 times per week, for 13 weeks. | Cognition (MMSE and Amsterdam Dementia Screening Test 6 (ADS 6)).  Behaviour (The BOP scale - Beoordelingsschaal voor Oudere PatieÈnten / Evaluation Scale for Elderly Patients, a Dutch adaptation of the Stockton Geriatric Rating Scale). | Medium |
| Yu and Kolanowski (2009) | Non-controlled before and after feasibility study | People with mild to moderate Alzheimer’s disease living in a retirement community, with resting heart rate (HR) less than 100 beats per minute, medical clearance from their primary care provider, and no physician-reported major neurological or affective disorders such as head trauma, multi-infarct dementia, or schizophrenia, unstable medical conditions such as congestive heart failure, previous heart attack or stroke within the previous 6 months; or severe chronic obstructive pulmonary disease.  (n=2) | An exercise program including warm-up activities (lower body stretches and marching in place at a casual pace), moderate-intensity cycling on a recumbent stationary cycle (60%–65% of maximal heart rate), and cool-down activities (slowing cycling and stretches).  From 25 rising to 45 minutes, 3 times per week, for 8 weeks. | Before and after intervention | Global cognition (MMSE).  Executive functioning (Stroop Neuropsychological test).  Ability to carry out activities of daily living (Instrumental Activities of  Daily Living scale (IADL) and Physical Self Maintenance Scale  (PSMS)).  Behavioural and psychological symptoms  of dementia (Columbia University Scale for Psychopathology  in Alzheimer’s Disease (CUSPAD)). | Low |

***Table 7: Key conclusions of included items***

| **Author(s), date and title of publication** | **Conclusions (quoted)** | **Recommendations (quoted)** | **Assessed quality of study** |
| --- | --- | --- | --- |
| Arakawa-Davies (1997) Dance / movement therapy and reminiscence: A new approach to senile dementia in Japan. | In Japan, dance/movement therapy is a new, non-medicinal approach to the treatment of senile dementia, which focuses on alleviating the physical, psychological and social suffering of patients, and in helping them maintain a healthy lifestyle in institutional settings.  Dance/movement therapy shows great promise in its application to the distinct Japanese cultural milieu, particularly in its role as an activator of reminiscence and life review. In contrast to prevailing trends in the West, in Japan, as in virtually all Asian societies, the elderly still play an important role as guardians of traditional customs and folklore and as the narrators (i.e., storytellers) of culture.... as it becomes more widely accepted, one of its most important and beneficial functions will be to act as a natural bridge between the reminiscing of the elderly and the narration of Japanese culture. | None made | Low |
| Baker et al (2010)  Effects of Aerobic Exercise on Mild Cognitive Impairment | Aerobic exercise is a cost-effective practice that is associated with numerous physical benefits.  The results of this study suggest that exercise also provides a cognitive benefit for some adults with MCI. Cognition enhancing effects of aerobic exercise were most pronounced for executive control tasks in women, an effect that was paired with increased insulin sensitivity and reduced circulating levels of cortisol and BDNF.   Six months of a behavioural intervention involving regular intervals of increased HR was sufficient to improve cognitive performance for an at-risk group without the cost and adverse effects associated with most pharmaceutical therapies.  The demands of the aerobic intervention are suited for a controlled trial, but may not be well-tolerated in less structured, less supervised population-based studies. | Further examination of associations between aerobic exercise–induced change in glucoregulation, HPA axis activity, and cognition may uncover mechanisms that could account for the sex bias in cognitive response.  Replication with a larger group of adults with MCI is essential. | Medium |
| Batman (1999)  The Effects of Therapeutic Aquatic Exercise on Patients with Alzheimer's Disease. | Data showed improvement in the overall behaviour ratings for those completing the therapeutic aquatic exercise compared to the control group. Subjects were observed to be less combative, require less medication, and sleep better at night (care-giver assessment, not observed by raters). Psychosocial benefits of group exercise experience may also improve quality of life (although no evidence offered re this conclusion). Those in experimental group increased muscle strength with exercise, for some leading to improvements in ambulation and less frequently expressed joint pain. | Findings warrant further research into aquatic exercise as an adjunct to medical and psychological treatment of Alzheimer's.  Current findings are not generalisable to wider community. Further studies need to have larger numbers, be undertaken at multiple sites, track variables that threaten internal validity, include full screenings for dementia at commencement, midpoint and end of study.  A second land-based exercise group would explore benefits of exercise alone.  The influence of group versus individual activity could also be explored. Care-givers could also meaningfully be involved in the exercise experience and benefits to them also evaluated. | Low |
| Binder (1995)  Implementing a structured exercise program for frail nursing home residents with dementia: Issues and challenges. | A group-based exercise program is feasible in institutionalized older adults with chronic dementia and because no expensive or specialized equipment is required, this program may also be readily adaptable to institutionalized older adults at risk for falls.  Those completing the intervention demonstrated significant improvements in hip extension and Romberg balance test scores and a decline in knee extension, but other measures of strength and gain showed no significant changes in performance.  After 8 weeks of exercise, there were no significant differences in performance between the exercise-only and exercise plus vitamin D supplement groups. | Research is needed to assess whether Cybex II dynamometry is able to produce a valid measurement of knee extension in people with dementia given the motivation and mental concentration required to produce maximal muscle contraction and whether alternatives, eg single repetition at maximum, provide increased validity in this context.  There should be a staff-to-participant ratio of at least 1:4 for exercise interventions with care home residents with dementia in order to achieve the appropriate exercise, and programs should optimally provide opportunities for exercise once or twice per day in order to assist with issues of compliance and decreased attention span.  The effects of vitamin D supplements on muscle strength need further investigation, as the study results are not conclusive due to methodological limitations. | Medium |
| Buettner and Fitzsimmons (2004)  Recreational therapy exercise on the special care unit: Impact on behaviors. | The groups had different levels of agitation at the start of the program - with the afternoon group significantly higher. There were differences in impact of providing morning versus afternoon exercise interventions for this group in terms of agitation and passivity. This contrasted with an earlier study by the authors where an afternoon exercise program for falls prevention was found to be effective. This underlined the importance of careful resident assessment for specific programs.  The intervention impacted on strength and flexibility scores for participants in the morning group but not the afternoon group (although this may have been attributable to health problems for some of the participants in this group), although changes were only significant at 4 weeks.  Time of day of recreational therapy offerings must match the needs of the participants to enhance functional outcomes and use staff time effectively. Mornings are superior to afternoons in terms of outcomes of this intervention. By afternoon residents may already have experienced numerous stresses that reduce their tolerance for activity and increase dysfunctional behaviour. | Interventions need to be tailored to the individual, who should be carefully assessed. Mornings are the most cost-effective time of day to provide exercise for function interventions. Care homes should consider changing their schedules to deliver this type of intervention in the mornings and move more relaxing activities to afternoons or evenings, or provide an afternoon recreational therapy relaxation program as a combined treatment approach. | Low |
| Buettner et al (2008) Evidence supporting exercise interventions for persons in early-stage Alzheimer's disease. | Studies considered demonstrated improvements primarily in cognition, physical and functional ability, depression and behavioural symptoms in exercisers compared with non-exercisers. Type of tested exercise has varied, including home-based aerobic/endurance activities, strength training, balance and flexibility training most common.  Only two studies using Tai Chi were found, but this form of exercise was seen as having additional benefits over other exercise forms for older adults. Aerobic exercise is also an exercise form with additional benefits and worthy of further exploration. | All recreational therapy programs for individuals with AD should include physical exercise, preferably aerobic exercise if it can be tolerated, but if not then exercises that are less strenuous yet promote strength, balance, and coordination, such as Tai Chi. | High |
| Burns et al (2008)  Cardiorespiratory fitness and brain atrophy in early Alzheimer's disease | There is a relationship between cardiorespiratory fitness and brain atrophy in the earliest clinical stages of AD. Higher fitness levels in early AD participants are associated with preserved brain volume (less brain atrophy) independent of age and dementia severity.  The cross-sectional study design limits the interpretation of the results.  Single point assessment of VO2(peak) means that data should be cautiously interpreted with respect to role of exercise in early AD. | Further longitudinal and interventional studies will be necessary to define the role of fitness in modifying the brain aging and AD process.  Further study of the relationship between fitness and vascular-related damage is needed to investigate further the possibility that a common underlying AD-related process may impact both brain atrophy and cardiorespiratory fitness. | Medium |
| Christofoletti et al (2008)  A controlled clinical trial on the effects of motor intervention on balance and cognition in institutionalized elderly patients with dementia | Six months of multidisciplinary or physiotherapeutic intervention improved the balance of institutionalized elderly people with dementia.  Global cognition did not improve with either intervention.  When the intervention was carried out on a multidisciplinary basis, the researchers observed an attenuation of cognitive decline in the domains of verbal fluency (measured by the Verbal Fluency Test) and executive functions (measured by the Clock Drawing Test).  Exercises applied in different contexts may have positive outcomes for people at a moderate stage of mixed dementia. | Other studies should be carried out to clarify whether other interventions could attenuate cognitive decline. | Medium-High |
| Dayanim (2009)  The Acute Effects of a Specialized Movement Program on the Verbal Abilities of Patients With Late-Stage Dementia. | The specialized movement therapy program has the ability to provide immediate acute effects on memory recall of patients with late-stage AD. After 20 minutes of movement therapy, participants appeared more organized in their speech abilities. This is an important finding as it suggests that some forms of physical activity—in this case movement therapy—can help mentally organize patients with AD as severe as those in the late stage of dementia in which they often have limited to no speech.  This program is simple and may be utilized for patients with AD by a caregiver or family member seeking a method of increased communication with their loved one. Although the duration of the cognitive effects was not examined, a moment of clarity between a patient with AD and a family member would certainly be an improvement and much welcomed change for the family member. | Future research should include a larger number of participants from various residents in hope to gain additional subgroup comparisons.  Future research should also investigate the duration of the increased object and colour recall following the movement session.  Future research should include a more comprehensive cognitive assessment before and after movement sessions. | Low |
| Dorner et al (2007) The effect of structured strength and balance training on cognitive function in frail, cognitive impaired elderly long-term care residents | Findings support the recommendation that structured strength and balance training should be implemented in long-term care facilities for frail elderly and very old persons.  Findings showed increased muscle strength, increased BMI and possibly improved cognitive function, which all contribute to improving independence and enhancing overall health and wellbeing of frail elderly persons. | Further work required to choose ideal composition of strength and balance motor components within the framework of a training program.  Longer term effects of training programs should be tested in further studies with larger sample sizes. | Medium |
| Edwards et al (2008) Effect of exercise on negative affect in residents in special care units with moderate to severe dementia. | There was a significant reduction in anxiety ratings from the beginning to the end of the exercise session when adjusted for level of participation, but no significant immediate effects on observer-rated anger or depression.  There were significant reductions in anxiety after 12 weeks of exercise after adjusting for level of participation. A significant decline was also noted in depressive affect after 12 weeks of exercise after adjusting for level of participation. No long-term effects were observed for anger.  Not all participants experienced positive short term or longer term effects on anxiety or depression.  This study demonstrated that it is feasible to conduct a chair-based exercise program with moderate to severe dementia residents of secured dementia units in long-term care facilities. These individuals were able to be encouraged to participate in the exercise routine through visual and verbal cueing. Two-thirds of the subjects participated more than 75% of the time.  An intervention that could reduce negative affect would not only impact the individual with negative affect, but also other residents, and in doing so, reduce work burden on staff.  The fact that exercise can successfully be performed in a group situation suggests the cost of administering the intervention may be less than the extra cost of care associated with symptoms of negative affect in the traditional manner. | Future research should also consider the influence of an exercise program on staff satisfaction and turnover.  Staff working with individuals with dementia listed anxiousness as very common. The ability to reduce negative affect and the resulting behaviours may positively influence staff satisfaction by reducing burden and possibly minimize staff turnover, resulting in consistent quality care and decreased healthcare cost. | Low-medium |
| Eggermont et al (2009a)  Hand motor activity, cognition, mood, and the rest–activity rhythm in dementia A clustered RCT | The only significant effect of hand movement sessions was on mood which was found to improve in a statistically significant way.  The authors suggest two reasons why this result was found. Firstly, dopamine levels are related to hand movements and to mood - so hand movements may increase dopamine levels and lead to improved mood - however dopamine levels not much affected by dementia so that may explain relatively minor improvements found.   Secondly: activation of the prefrontal cortex, caused by hand movements will lead to a diminishment of the corticotropin releasing hormone neurons of the hypothalamo–pituitary–adrenal-axis, and so to an improvement of mood. | The main finding is encouraging since hand motor activity is a type of activity that can be applied at a large scale. In view of the expanding population of older nursing home residents with dementia, studies focussing on the development and fine-tuning of suitable intervention programs in this population are warranted.  The observation of a potential mirror neuron system in humans offers possibilities for innovative intervention studies. In very frail older persons with dementia in which hand function is decreased, the observation of hand movements and the subsequent activation of brain areas may be a suitable intervention.  All these future research efforts may offer a valuable contribution to the development of programs concerning useful day time activity in this frail older population. | High |
| Eggermont et al (2009b)  Walking the line: a randomised trial on the effects of a short term walking programme on cognition in dementia. | No positive effects on cognition were found, and treatment outcome was not influenced by ApoE genotype.  The current study does not support the notion that physical activity benefits cognition in older nursing home residents with moderate dementia, who suffer from cardiovascular disease. The precise influence of these potential modifiers of treatment outcome after physical activity remains to be elucidated in further studies. | In future research, blood pressure and heart rate should be measured to acquire a general idea of the level of physical activity as effects of aerobic and anaerobic activity have been shown to be different.  Future studies should take into account the presence of vascular co-morbidities. The presence of vascular disease may have limited the potentially positive effects of walking on cognition as the increase in cerebral perfusion that is normally seen after exercise may have been reduced.  Future studies should have longer duration, as there may be benefits of walking that manifest in the longer term through benefits to the cardiovascular system.  Information on the specific subtype of dementia diagnosis might be informative. | Medium |
| Francese, Sorrell and Butler (1997)  The effects of regular exercise on muscle strength and functional abilities of late stage Alzheimer's residents. | Clinicians should further study the value of exercise for residents with senile dementia of the Alzheimer's type. Within the intervention group some participants gained enough muscle strength to stand and transfer with assistance having previously required help from two staff members. The authors also noted that there was a significant change in the staff attitudes to working with the residents in the experimental group, with staff being more positive about their approach to activities of daily living care for these residents after the study than before. | There is a need for continued research on people living in nursing facilities with senile dementia of the Alzheimer's type.  It is important to understand relationships of cognitive decline with other functions of the body in order to assist residents with senile dementia of the Alzheimer's type to gain the maximum use of physical functioning for as long as possible.  It is also important for caregivers and staff to understand the disease progression and the physical capabilities of residents, as this can help reduce injury to staff and residents and assist staff in maintaining physical functioning and improving the quality of life for residents. | Low |
| Friedman and Tappen (1991)  The effect of planned walking on communication in Alzheimer's disease | A planned walking program can improve Alzheimer's patients' ability to communicate, in contrast to conversation only which does not result in significant improvement.  The planned walking group increased average COS score from 18.40 to 22.93 (out of a maximum 32), whereas the conversation only group had a slightly decreased COS score. The planned walking group increased average CAS score from 45.40 to 59.00 (out of a maximum 93), whereas the conversation only group's score increased only marginally score. It may be necessary to stimulate expressive and receptive regions of the brain in addition to the motor cortex to achieve improvements in communication performance. | The addition of a no-treatment group would have increased control of endogenous variables.  The addition of a walking without conversation group would have clarified the different roles of walking and conversation in the study results.  The simplicity and cost effectiveness of the intervention provide an adaptable approach for improving the function of communication for those with Alzheimer's, however the assumption that it would not be necessary to use highly trained personnel to implement it should be tested.  It might also be possible for the intervention to be delivered to small groups (2-3 people) provided that the staff member communicates with all those walking. | Medium |
| Hernandez et al (2010) Effects of physical activity on cognitive functions, balance and risk of falls in elderly patients with Alzheimer’s dementia. | Patients with AD who participated in the program of systematic physical activity improved maintenance of cognitive functions, and performance in balance and decreased risk of falls. In contrast, patients with AD who did not take part in the physical activity program showed a greater decline in cognitive function, reduction in balance and increased risk of falls. Physical activity may represent an important non-pharmacological contribution to diminish the rate of cognitive and motor decline due to the progression of the disease. | Regarding the motor tests used to evaluate patients with AD, the stimulation and instruction during the tests are extremely important. However, the literature does not elucidate this kind of stimulation, highlighting the need to develop and adapt techniques to instruct elderly patients with AD in these tests so that the quality of their performance is not affected during the tests. Therefore, during the intervention protocol, clear, objective and repetitive instructions were designed to guide the participants in performing the exercises. | Low |
| Heyn, Abreu and Ottenbacher (2004) The effects of exercise training on elderly persons with cognitive impairment and dementia: a meta-analysis | Exercise was associated with statistically significant positive treatment effects in older patients with dementia and cognitive impairments.  Meta-analysis suggested a medium to large treatment effect for health-related physical fitness components, and an overall medium treatment effect for combined physical, cognitive, functional and behavioural outcomes.  The results provide preliminary evidence for the effectiveness of exercise treatments for persons with dementia and related cognitive impairments. | Further research is needed to document safety, preparedness and appropriateness of exercise program for older people with dementia.  The impact of the environment on increasing levels of physical activity in institutionalised older adults should be explored further.  Research is needed to determine how to motivate persons with Alzheimer's disease to exercise regularly, and also to engineer and adapt exercises to fit various health needs.  Future studies should involve multicentre trials with rigorous experimental controls to help resolve issues with study design such as absence of blinding procedures and small sample sizes. | High |
| Holliman, Orgassa and Forney (2001) Developing an interactive physical activity group in a geriatric psychiatry facility. | Participants exhibited less disruptive behaviour and more positive behaviours during the group sessions. Participants did not display less disruptive behaviour outside the group.  Participants looked forward to the intervention and change their behaviour as soon as they were in the intervention setting, indicating that positive experiences have to be reinforced and repeated with this population to make a difference. Reinforcement needs to be incorporated into the daily schedule of residents.  An interactive physical intervention is relatively easy to facilitate. | The current study had a very short time period, and the authors recommend that interventions should be implemented for much longer periods of time, at least 6 to 10 weeks, to see if the positive responses are consistent phenomenon.  Activity blocks should be expanded for people with dementia in residential settings in order that the positive experiences associated with physical activity interventions can be reinforced and repeated regularly.  Staffing schedules and staffing allocation should be changed to allow for increased recreational activities. Prioritising frequent meaningful activities for older people in residential settings is key to improving quality of life. | Medium |
| Lange-Asschenfeldt and Kojda (2008)  Alzheimer’s disease, cerebrovascular dysfunction and the benefits of exercise: From vessels to neurons | 'Next to upholding neuronal plasticity, regular exercise may counteract AD pathophysiology by building a vascular reserve'.  Exercise potently enhances not only neuronal but also vascular plasticity. Thus, by counteracting the ever increasing vascular stress in late-life, exercise may heighten the threshold for the manifestation of cardiovascular as well as neurodegenerative diseases like AD where vascular impairment has a strong synergistic effect on the specific pathophysiology. As long as a cure for the disease is not in sight, prevention will remain a major focus in combating it.  By antagonizing vascular oxidative stress and VRFs from early midlife on, exercise appears to be a promising tool in the prevention of AD. | The authors seem to recommend regular physical exercise in mid-life. | Medium |
| Littbrand et al (2006) A high-intensity functional weight-bearing exercise program for older people dependent in activities of daily living and living in residential care facilities: Evaluation of the applicability with focus on cognitive function. | A high-intensity functional weight-bearing exercise program is applicable for use, regardless of cognitive function, among older people who are dependent in ADL, living in residential care facilities, and have an MMSE score of 10 or higher.  An important factor for the high attendance rate in this study, especially for the participants with severe cognitive impairment, was probably that reminders were used.  The approvals from the participants’ physicians prior to the study were probably important for the participants’ safety as well as the supervision by physical therapists who were experienced in working with frail older people and adjusted the exercises for each session depending on changes in the participants’ health status. | No additional points noted. | High |
| Palo-Bengtsson and Ekman (2002) Emotional response to social dancing and walks in persons with dementia | The synchronous movement in dancing created an easy atmosphere, which in turn created mutual tenderness and communion between persons with dementia and their caregivers. This atmosphere was less easy to achieve in walks.  Movement in both activities was connected with caregivers' active engagement with participants, constant encouragement, and sensitive responses to participants' needs.  Communication prompted by dance and walking activities was connected to emotional willingness to form new social contacts. | Interventions involving body movements should become an organised part of the care process to encourage appropriate emotional reactions in people with dementia.  Musical events with opportunities to dance should be organised more often than those without to provide people with dementia with the opportunity to move and to use their bodies to articulate emotions.  For people with dementia in wheelchairs, a short walk outside of the chair with a supportive caregiver may be better than a long walk confined to the wheelchair in terms of maximising the potential for a positive emotional response. | Low |
| Rolland et al (2007) Exercise program for nursing home residents with Alzheimer's disease: a 1-year randomized, controlled trial | This study provides evidence that a moderate exercise program conducted twice a week significantly slows, by approximately one-third, the progressive deterioration in ability to perform ADLs in people with AD living in nursing homes. With a treatment difference of 0.39 points (about a 6.7% benefit relative to the control group) at the study end point, this ADL change corresponds to a small but clinically meaningful benefit of the exercise program.  This intervention was associated with improvement in mean walking speed but had no significant effect on nutritional status, behavioural disturbance, or depression measures.  The exercise program was safe.  The intensive exercise approach of this study may be more effective than some less intensive exercise programs but may not be practical in some nursing home settings in the long term. | Whether offering more exercise sessions would have led to better results warrants further investigation, although additional sessions may result in lower adherence.  Future work should focus on efforts to improve adherence. | Medium-High |
| Scherder et al (2007) Physical activity and cognition in Alzheimer's disease: Relationship to vascular risk factors, executive functions and gait. | Results from epidemiological studies indicating positive relationship between physical activity and cognition suggest that clinicians working with people with dementia should focus on levels of physical activity as well as cognition.  More RCTs of effects of physical activity on cognition are required. Studies reviewed (with less rigorous designs) show physical activity may improve global cognitive functioning, but effects on specific cognitive functions are inconsistent.  Lack of positive effects of physical activity on cognition might be due to presence of cerebrovascular disease in AD, causing disturbances in executive function and gait which lower levels of physical activity and prevent participants from exercising at sufficient intensity to achieve cognitive benefits.  In addition to disturbances in executive function and gait, vascular risk factors such as hypertension may provide additional explanation for negative effect of physical activity on cognition for those with AD, as is suggested that blood supply to muscles may be at expense of cerebral perfusion.  There is a paucity of studies examining effects of (non)pharmacological interventions on executive functions and gait in people with AD. | Physical activity programmes aiming to enhance cognition should consider disturbances in executive function and gait and cardiac condition of participant to maximise efficacy on physical functioning and hence cognition. | Medium-Low |
| Steinberg et al (2009) Evaluation of a home-based exercise program in the treatment of Alzheimer's disease: the Maximizing Independence in Dementia (MIND) study | Significantly improved performance was found on a hand function task predictive of ADL performance, with a trend for improved performance on a test of lower extremity strength. No differences were found in the amount of time spent in vigorous physical activity or in 8-foot timed walking speed.  Among the secondary outcome measures, no benefits were noted; if anything, there was a suggestion of both worse depression and lower quality of life ratings with exercise. Increased neuropsychiatric burden was not mirrored in either the NPI total score or depression sub-score.  A caregiver-supervised home-based exercise intervention can be can be performed with acceptable compliance | The possibility that exercise in AD may be associated, at least in some patients, with increased distress warrants further exploration.  Whilst no serious adverse events were attributed to participation in the study, and neither group appears favoured regarding such events, statistical inference is limited by the sample size and the higher frequency of total adverse and cardiovascular events in the exercise group warrants further study.  Given the limited efficacy to date of pharmacotherapies for AD, research is needed to investigate the effects of a feasible exercise intervention in a variety of settings including assisted living and long term care, as well as to address whether it may delay need for placement in a higher level of care. | High |
| Van de Winckel et al (2004) Cognitive and behavioural effects of music-based exercises in patients with dementia | Daily music-based seated dance sessions for three months led to significantly higher scores on the total MMSE score in patients with dementia. The effect size of 0.5 suggested that this outcome could be considered clinically relevant. It could represent a worthwhile approach to slow down the progression of disability.  The exercise group also improved significantly on the `category fluency’ test in ADS 6 after 6 and 12 weeks, when compared with the control group. In a category fluency test, patients are required to say words that belong to a same semantic category (for example `animals’). This skill requires temporal lobe-mediated semantic knowledge and is more impaired in patients with Alzheimer’s disease, who are predominantly affected in medial temporal and posterior cortical regions.  According to the results on the BOP scale, there are no significant behavioural effects resulting from the treatment.  The intervention is feasible for staff to deliver with minimal training, and does not require costly exercise equipment.  Mimicking the movement appears to be the most important cue. When repetitive verbal cueing was given without visual aid, the patients continued with the last shown movement. | No additional points noted. | Medium |
| Yu and Kolanowski (2009) Facilitating aerobic exercise training in older adults with Alzheimer’s Disease. | Engaging older adults with AD in aerobic exercise is important, because aerobic exercise training improves physical functioning and has the potential to alleviate AD symptoms.  The aerobic exercise protocol developed for older adults with mild to moderate AD appears in early pilot testing to be safe, feasible, and easy to implement.  Proactive and respectful communications with primary care practitioners is expected to facilitate recruitment and medical clearance.  The Borg scale (provides subjective rating of exercise intensity) offers unique information by participants but could be interruptive. HR monitoring (not used in present study) is an excellent way of objectively monitoring exercise intensity and is not invasive or interruptive.  An exercise physiologist should be included as a team member in future studies. Exercise physiologists can help further quantify the exercise prescription, select measures for physical functioning, and troubleshoot any problems arising from exercise training. | Aerobic exercise protocol requires additional testing with a larger group of participants.  Further studies are necessary that examine the cognitive, functional, and behavioural outcomes of this aerobic exercise program in older adults with AD.  Future studies should measure cognitive, executive functioning, functional, and behavioural outcomes with adequate power and longitudinal follow-up. | Low |

***Table 8: Survey results***

Total number of survey responses: 73

| **How did you hear about this research project?** | per cent | number |
| --- | --- | --- |
| DSDC newsletter | 47% | 34 |
| PAHA newsletter | 1% | 1 |
| Other* | 52% | 38 |
| Total number of respondents |  | 73 |

*Others included: manager, emails, internet search, 2 mentioned Journal of Dementia Care

| **Job titles of respondents** | per cent | number |
| --- | --- | --- |
| Activities coordinator | 24% | 18 |
| Occupational therapist | 14% | 10 |
| Research manager | 1% | 1 |
| Care home manager/deputy | 24% | 18 |
| Community mental health nurse | 3% | 2 |
| Physiotherapist | 4% | 3 |
| Care worker/senior care worker | 3% | 2 |
| Manager (unspecified/varied) | 16% | 11 |
| Lecturer/practitioner (nursing) | 3% | 2 |
| Day care organiser | 3% | 2 |
| Home care organiser | 1% | 1 |
| Housing support coordinator | 1% | 1 |
| Independent trainer | 1% | 1 |
| Mental health clinical support worker | 1% | 1 |
| Total number of respondents |  | 73 |

| **Gender of respondents** | per cent | number |
| --- | --- | --- |
| Female | 89% | 64 |
| Male | 11% | 9 |
| Total number of respondents |  | 73 |

| **Age of respondents** | per cent | number |
| --- | --- | --- |
| under 20 | 0% | 0 |
| 21-30 | 11% | 8 |
| 31-40 | 19% | 14 |
| 41-50 | 34% | 25 |
| 51-60 | 29% | 21 |
| 61-70 | 7% | 5 |
| 0ver 70 | 0% | 0 |
| Total number of respondents |  | 73 |

| **Role of respondents with activity programme (tick all that apply)** | per cent | number |
| --- | --- | --- |
| Design of programme | 51% | 37 |
| Commissioning of programme | 19% | 14 |
| Management of programme | 62% | 45 |
| Delivery of programme | 63% | 46 |
| Other | 6% | 4 |
| Total number of respondents |  | 73 |

| **Specialist training in physical activity for people with dementia** | per cent | number |
| --- | --- | --- |
| Yes | 41% | 30 |
| No | 59% | 43 |
| Total number of respondents |  | 73 |

There seem to be many different training courses that have been undertaken by respondents. These include specific training on particular approaches to physical activity for people with dementia (e.g. Tai Chi for dementia, Fitness leader training at Stirling University), physical activity for older people (e.g. ‘seated exercise’ four day course, Active Fife training for older people, Alzheimer’s Society training, jabadao and SONAS training) and other more general training courses that relate to care and activities for people with dementia.

| **Where are programmes provided? (tick all that apply)** | per cent | number |
| --- | --- | --- |
| Community based health services | 24% | 17 |
| Hospital health services | 13% | 9 |
| Community based social care | 13% | 9 |
| Residential or nursing care | 59% | 41 |
| Other* | 7% | 5 |
| Total number of respondents |  | 70 |

*Others included: day care (4), peer led activities and housing support

| **Within which sector are activities provided?** | per cent | number |
| --- | --- | --- |
| Private | 31% | 22 |
| Public | 59% | 41 |
| Not-for-profit | 19% | 13 |
| Total number of respondents |  | 70 |

| **Location of programme** | per cent | number |
| --- | --- | --- |
| England | 27% | 19 |
| Northern Ireland | 4% | 3 |
| Scotland | 60% | 42 |
| Wales | 0% | 0 |
| Other* | 9% | 6 |
| Total number of respondents |  | 70 |

*Others: Australia, Malta, 3 in Ireland

| **Who delivers the activity programmes? (tick all that apply)** | per cent | number |
| --- | --- | --- |
| Occupational therapist | 25% | 17 |
| Physiotherapist | 17% | 12 |
| Nurse | 19% | 13 |
| Nursing assistant | 10% | 7 |
| Social worker | 3% | 2 |
| Social care worker/assistant | 33% | 23 |
| Activities coordinator | 54% | 37 |
| Volunteer | 17% | 12 |
| Other* | 15% | 10 |
| Total number of respondents |  | 69 |

*Other: carers, students, OT support staff, care assistants, Tai Chi Instructor, dance artist, physiotherapy assistant, peers, ‘We do not yet have an activities coordinator’, people who provide the service as their business, locality links officer, SW dept, healthcare assistant under supervision of the OT, OT assistant.

| **Age range of activity programme participants (tick all that apply)** | per cent | number |
| --- | --- | --- |
| below 50 | 6% | 4 |
| 51-55 | 12% | 8 |
| 56-60 | 17% | 12 |
| 61-65 | 46% | 32 |
| 66-60 | 75% | 52 |
| 71-75 | 87% | 60 |
| 76-80 | 94% | 65 |
| 81 and above | 90% | 62 |
| Total number of respondents |  | 69 |

| **Who participates in the activities (gender)?** | per cent | number |
| --- | --- | --- |
| All men | 4% | 3 |
| All women | 1% | 1 |
| Mixed | 94% | 65 |
| Total number of respondents |  | 69 |

| **Who participates in the activities (age)? (tick all that apply)** | per cent | number |
| --- | --- | --- |
| People with dementia over 65 only | 6% | 4 |
| People with dementia of any age only | 18% | 13 |
| People with dementia under 65 only | 3% | 2 |
| Anyone over 65 including people with dementia | 34% | 24 |
| Anyone under 65 including people with dementia | 3% | 2 |
| People of any age including people with dementia | 42% | 28 |
| Other* | 8% | 5 |
| Total number of respondents |  | 69 |

*Others: These included services that have different age cut offs (such as 50 or 60) and two projects for younger people that allow people to remain beyond 65 if that want to.

| **Are there eligibility criteria?** | per cent | number |
| --- | --- | --- |
| Yes | 33% | 23 |
| No | 67% | 46 |
| Total number of respondents |  | 69 |

Criteria relate either to the service (must attend or be referred to a particular service, must be within a local authority area) or to the individual (must be assessed to have potential to benefit, must be interested in programme, ability to participate, must have dementia and so on).

| **Where do participants live?** | per cent | number |
| --- | --- | --- |
| Community | 39% | 27 |
| Sheltered housing | 23% | 16 |
| Care home | 62% | 43 |
| Hospital | 13% | 8 |
| Total number of respondents |  | 69 |

| **Is programme open for carers?** | per cent | number |
| --- | --- | --- |
| Yes | 65% | 45 |
| No | 35% | 24 |
| Total number if respondents |  | 69 |

| **How often do participants attend?** | per cent | number |
| --- | --- | --- |
| Daily | 30% | 20 |
| Weekly | 51% | 34 |
| Monthly | 2% | 1 |
| Other* | 18% | 12 |
| Total number of respondents |  | 67 |

***Other: ‘** depends on the individual (person centred approach)’, ‘depends on day care attendance’ and other timing options such as twice weekly.

| **Where does programme take place? (tick all that apply)** | per cent | number |
| --- | --- | --- |
| Clinical setting | 13% | 9 |
| Community building | 8% | 5 |
| Care home | 55% | 37 |
| Day centre | 27% | 18 |
| Individual’s home | 5% | 3 |
| Park | 8% | 5 |
| Other outside location* | 21% | 14 |
| Other indoor location* | 10% | 7 |
| Total number of respondents |  | 67 |

*Other locations include: garden of care setting, out in the community, workshop, allotment and hospital grounds.

**How many participants in each session?**

Between 1 and 60

Average: 8.5

**Date programmes started:**

Earliest start date: 1991

Average length of programme: 4 years

| **Funding for programme** | per cent | number |
| --- | --- | --- |
| Provided in care home as part of normal service | 26% | 17 |
| Provided in care home plus fundraising top-up | 6% | 4 |
| Fundraising | 9% | 6 |
| NHS funding | 22% | 14 |
| Budget from organisation plus fundraising | 5% | 3 |
| Private organisation | 3% | 2 |
| Joint NHS and LA funding | 3% | 2 |
| Local authority service | 9% | 6 |
| Fee for service users | 6% | 4 |
| N/A | 11% | 8 |
| Total number of respondents |  | 66 |

| **Is there a cost to service users?** | per cent | number |
| --- | --- | --- |
| Yes | 14% | 9 |
| No | 86% | 57 |
| Total number of respondents |  | 66 |

| **Duration of funding** | per cent | number |
| --- | --- | --- |
| Ongoing | 53% | 24 |
| N/A | 24% | 11 |
| Unsure | 9% | 4 |
| Yearly review | 4% | 2 |
| Individual basis | 2% | 1 |
| Set funding time (three months or less) | 4% | 2 |
| Varied | 2% | 1 |
| Total number of respondents |  | 45 |

| **Activities mentioned in programme descriptions**  **NB. Many programmes include more than one activity** | Number of participants mentioning activity |
| --- | --- |
| Seated exercise with music | 15 |
| Walking | 13 |
| Exercise programme or group | 13 |
| Dancing | 11 |
| Bowling/skittles/curling | 11 |
| Gardening | 9 |
| Seated exercise (no music mentioned) | 6 |
| Quigong/Tai Chi | 6 |
| Golf/driving range/putting | 5 |
| Wii sports | 5 |
| Billiards/snooker | 3 |
| Keep fit classes/aerobics | 3 |
| Parachute game | 3 |
| Time in garden | 2 |
| Cookery | 2 |
| Chair football | 2 |
| Outings | 2 |
| Housework | 2 |
| Sailing/speed boats | 2 |
| Stretching exercises | 2 |
| Singing with actions | 1 |
| Weight training | 1 |
| Fishing | 1 |
| Reminiscence (acting out use of old objects) | 1 |
| Table net ball | 1 |
| Darts | 1 |
| Exercise with music | 1 |
| Otago | 1 |
| Exercise bike | 1 |
| Tasks of daily living | 1 |
| Swimming | 1 |
| Horse riding | 1 |
| Woodwork | 1 |
| Bikes/trikes | 1 |
| SONAS | 1 |
| Number of respondents | 62 |

| **Thinking behind programme (tick all that apply)** | per cent | number |
| --- | --- | --- |
| Fun/enjoyment | 27% | 17 |
| Social interaction | 26% | 16 |
| Wellbeing (and health in general terms) | 19% | 12 |
| Mobility and posture | 13% | 8 |
| Self esteem/confidence | 11% | 7 |
| Physical and mental stimulation | 11% | 7 |
| Communication | 6% | 4 |
| Improve/retain skills | 6% | 4 |
| Falls prevention | 6% | 4 |
| Relationships with staff and carers | 5% | 3 |
| Strength and fitness | 5% | 3 |
| Increase independence | 3% | 2 |
| Other  Including:  Maximise potential, Fulfilling wishes, Alleviating boredom, Stimulate appetite, Manage challenging behaviour, Learning new activities, Improve sleep, Fewer visits to GP, Improve skin integrity, Assessment of abilities by staff | 16% | 10 |
| Total number of respondents |  | 62 |

| **Has an evaluation taken place?** | per cent | number |
| --- | --- | --- |
| Yes | 42% | 25 |
| No | 58% | 35 |
| Total number of respondents |  | 60 |

| **Who undertook the evaluation?** | per cent | Number* |
| --- | --- | --- |
| Actors internal to the organisation delivering the programme | 78% | 18 |
| Actors external to the organisation delivering the programme | 22% | 5 |

*Two respondents who said an evaluation had taken place did not give information about it.

**Evaluation findings:**

Little detail is given by respondents on the findings from the evaluations undertaken. The following outcomes were mentioned with the number of respondents citing these given in brackets.

- Increased socialisation (1)
- More leisure participation (1)
- Plans to expand programme due to success (1)
- Respite for carers (1)
- Increased functioning for participants (1)
- Positive feedback from participants (6)
- Improved balance/coordination (3)
- Improved muscle power (1)
- Confidence/self worth increased (5)
- Anxiety reduced (1)
- Living independently for longer (1)
- Improvement in wellbeing (3)

**Comments on evaluations:**

Respondents reflected in different ways on the evaluations that had taken place. There were varied comments made but these fell into two common themes:

- The need for more formal evaluation of programme/services – evaluation may currently take place informally but it is not written down.
- Often the findings showed that participants’ responses to activity programmes are positive.

**Care homes**

We looked more carefully at the data from care homes to see if they were providing activities in different ways to other settings.

Care homes tended to involve older people (those over 60) in their physical activities, similar to hospital settings. Community settings and sheltered housing provided for younger people.

Care homes were more likely to utilise activity coordinators to provide activities, while in other settings occupational therapists are the most common practitioners involved.

Care homes did include participants from other settings such as the community and sheltered housing in their physical activities, 18.6% of care home respondents included people from both these settings in their activities. Care homes also open their activities to carers, 86% of respondents stated that carers could attend the sessions.

Care homes were providing physical activity on a regular basis with 92.9% of respondents providing activity once a week or more often. One respondent was providing activity twice daily.

We also considered if care homes were undertaking a different range of activities compared to the other settings. What we found was that particular activities took place almost exclusively in care homes, such as seated exercise with music, while other activities were common in all settings, such as dancing, while other activities were relatively rare in care homes, such as gardening. These findings are illustrated in the following table:

| **Activities in care homes compared with other settings** | | |
| --- | --- | --- |
| Activity | % of responses from care homes | % of responses from other settings |
| Seated exercise with music | 87% | 13% |
| Walking | 77% | 23% |
| Quigong/Tai Chi | 67% | 33% |
| Bowling/skittles/curling | 66% | 36% |
| Exercise programme or group | 62% | 39% |
| Wii games | 60% | 40% |
| Dancing | 55% | 46% |
| Golf/driving range/putting | 20% | 80% |
| Gardening | 11% | 89% |
